# Atezolizumab plus bevacizumab and chemotherapy as first-line therapy for cervical cancer: a cost-effectiveness analysis in the US

**DOI:** 10.3389/fimmu.2024.1481584

**Published:** 2024-11-27

**Authors:** Yingtao Lin, Cijuan Li, Chang Wang, Jian Chen, Yuanqing Huang

**Affiliations:** ^1^ Clinical Medical Research Center, Clinical Oncology School of Fujian Medical University, Fujian Cancer Hospital, Fuzhou, Fujian, China; ^2^ Department of Comprehensive Surgery, Fujian Maternity and Child Health Hospital, College of Clinical Medicine for Obstetrics and Gynecology and Pediatrics, Fujian Medical University, Fuzhou, Fujian, China; ^3^ Department of Medical Oncology, Clinical Oncology School of Fujian Medical University, Fujian Cancer Hospital, Fuzhou, Fujian, China; ^4^ Department of Gynecological-Surgical Oncology, Clinical Oncology School of Fujian Medical University, Fujian Cancer Hospital, Fuzhou, Fujian, China; ^5^ Fujian Maternity and Child Health Hospital, College of Clinical Medicine for Obstetrics and Gynecology and Pediatrics, Fujian Medical University, Fuzhou, Fujian, China

**Keywords:** atezolizumab, cervical cancer, metastatic, cost-effectiveness, BEATcc trial

## Abstract

**Objective:**

Medication is the predominant therapy for advanced cancers. However, the use of novel anticancer medications is a major contributor to disease-related financial hardships. Recently, numerous countries have mandated the pharmacoeconomic assessments of novel oncological agents to mitigate patient financial risks and optimize resource allocation. The present study evaluated the cost-effectiveness of adding atezolizumab to standard therapy (atezolizumab plus bevacizumab [BC]) for metastatic, persistent, and recurrent cervical cancer from the perspective of US healthcare payers, with the aim of supporting policymaking and promoting the rational use of healthcare resources.

**Methods:**

Using clinical efficacy and safety data from the BEATcc clinical trial, in addition to cost and utility values from publicly available databases and published literature, a partitioned survival model over a 20-year lifetime horizon was developed to assess the cost-effectiveness of atezolizumab plus bevacizumab and chemotherapy (ABC) versus BC. The primary output of the model was the incremental cost-effectiveness ratio (ICER) and sensitivity analyses were performed to assess its robustness.

**Results:**

At both 20 and 4.5 y of time horizon, ABC therapy showed poor cost-effectiveness, with ICER of $193926.48/QALY and $168482.26/QALY, respectively, which were higher than the $150,000/QALY willingness-to-pay threshold. One-way sensitivity analysis showed that the price of atezolizumab had the most significant impact on the model results. When the price of atezolizumab was reduced by 10%, ABC changed from being not cost-effective to cost-effective (ICER = $121531.24/QALY). Probabilistic sensitivity analysis showed a 32.6% probability that ABC would be cost-effective, which increased to 58.6% when the price of atezolizumab was reduced by 10%.

**Conclusions:**

For patients with metastatic, persistent, and recurrent cervical cancer in the US, ABC was not as cost-effective as BC. Appropriate price reduction (10%) is recommended for atezolizumab to improve cost-effectiveness of ABC therapy.

## 1 Introduction

Although the incidence of cervical cancer has decreased in several areas worldwide owing to population-based human papillomavirus vaccination and molecular screening tests (1, 2), it remains a major disease burden (3). According to recently published statistics, there were 661,021 new cases and 348,189 deaths resulting from cervical cancer in 2022, ranking fourth in terms of both incidence and mortality among women (4). Of all patients, 49.7% were diagnosed with metastatic disease (5).

Platinum-based chemotherapy (cisplatin monotherapy [preferred] or cisplatin/fluorouracil combination) has been the standard of care for cervical cancer stages IB3, II, III, and IVA, although therapeutic options for advanced disease remain limited. For patients with recurrent or metastatic disease, systemic therapy with or without radiation has been the cornerstone of treatment, with the platinum-paclitaxel combination being the preferred first-line systemic regimen prior to the advent of anti-angiogenic agents and immune checkpoint inhibitors (ICIs) (6). Recent research has fundamentally transformed the therapeutic landscape of advanced cervical cancer by showing that the integration of anti-angiogenic agents and ICIs with conventional chemotherapy significantly improves survival outcomes in patients with persistent, recurrent, or metastatic diseases (7, 8). The current National Comprehensive Cancer Network (NCCN) guidelines recommend pembrolizumab plus chemotherapy (with or without bevacizumab) for PD-L1-positive patients, and chemotherapy plus bevacizumab as the first-line therapy for patients with advanced cervical cancer. For patients who experience disease progression following first-line treatment, pembrolizumab (PD-L1-positive) or cemiplimab is recommended as a second-line option (6).

A recently published BEATcc phase III randomized clinical trial investigated the efficacy and safety of adding atezolizumab (ABC regimen) to bevacizumab plus chemotherapy (BC regimen). Significant improvements in both progression-free survival (PFS) (13.7 versus 10.4 mo; hazard ratio [HR] = 0.62; 95% confidence interval [CI]: 0.49–0.78) and overall survival (OS) (32.1 versus 22.8 mo; HR = 0.68; 95% CI: 0.52–0.88) were observed in the ABC group (9). Atezolizumab is an ICI that targets PD-L1 expressed on cancer cells and has been approved by the Food and Drug Administration for the treatment of alveolar soft tissue sarcoma, hepatocellular carcinoma, melanoma, and lung cancer (10). The promising results of the BEATcc trial suggest a potential novel standard for first-line treatment of metastatic, persistent, and recurrent cervical cancer.

Irrespective of the significant efficacy of innovative therapies, their high cost presents significant challenges. For instance, pembrolizumab is priced at approximately $57/mg (11), with a standard dosing regimen of 200 mg/3 wk, resulting in annual treatment costs of up to $200,000. Irrespective of health insurance coverage, patients often incur substantial out-of-pocket costs. Faraj et al. (12) reported that pharmaceutical costs were the primary driver of cancer-related financial toxicity among patients with advanced malignancies, prompting critical discussions regarding pharmaceutical pricing strategies (13). To reduce the financial burden on patients and optimize healthcare resource allocation, the U.S. healthcare system has increasingly incorporated health-economic assessments since 2000 to inform decisions regarding drug adoption, pricing, and clinical utilization. Previous studies have shown the cost-effectiveness of pembrolizumab combined with chemotherapy in patients with advanced cervical cancer (14, 15). Considering the significant efficacy of ABC therapy, which is expected to become the new first-line therapy for patients with advanced cervical cancer, evaluations of the cost-effectiveness of ABC therapy compared to that of BC therapy are required. The present study aimed to conduct a cost-effectiveness analysis comparing ABC and BC therapies for the treatment of metastatic, persistent, and recurrent cervical cancer from the perspective of the U.S. healthcare system. The findings will contribute to the rational pricing of novel anticancer therapies, while providing evidence-based guidance for policymakers to optimize healthcare resource allocation and clinical practice guidelines.

## 2 Methods

Based on the BEATcc clinical trial and Checklist for Economic Evaluation Reporting Standards Statement (**Supplementary Material, Table S1**), a partitioned survival model (PSM) was used to analyze the cost-effectiveness of ABC.

### 2.1 Patients and intervention

The target population of this study matched the BEATcc clinical trial: adults (≥18 y) with measurable, metastatic, persistent, or recurrent cervical cancer not amenable to curative surgery or radiation, squamous cell carcinoma, or adenocarcinoma subtype with GOG or Eastern Cooperative Oncology Group performance status of 0 or 1. Enrolled patients were stratified based on previous concomitant chemoradiation, histological subtype, and platinum backbone, and were randomly assigned in a 1:1 ratio to the BC or ABC arm. The baseline characteristics of the enrolled patients are presented in **Supplementary Table S2**. Ethical approval was waived because the study population was based on patient characteristics published in the BETAcc clinical trial (the trial adhered to ethical standards, was approved by the Ethics Committee, and was publicly available) (9).

In a 3-week cycle, patients received bevacizumab in the BC arm and bevacizumab plus atezolizumab in the ABC arm until disease progression; crossover from the BC arm to the ABC arm at progression disease (PD) was not permitted. In addition, all patients received cisplatin (50 mg/m²), carboplatin (area under the curve, 5 mg/m²), or paclitaxel (175 mg/m²) for a maximum of six cycles. The body surface areas of the patient were calculated from body measurements of adult females published by the National Centre for Health Statistics (16). The treatments were administered on the first day of each cycle and continued until disease progression, unacceptable toxicity, patient withdrawal, or death, whichever occurred first. Based on clinical trials and NCCN guideline recommendations (6, 9), patients with PD were assumed to receive ICIs or chemotherapy as second-line treatment, whereas those who did not receive second-line treatment were assumed to receive the best supportive care. According to a clinical trial, the proportions of patients who received second-line treatment with ICIs, chemotherapy, and best supportive care were 3%, 51%, and 46% in the ABC group, compared with 33%, 25%, and 42% in the BC group. Patients who received ICIs in each group were treated with equal probability with either pembrolizumab or cemiplimab, and those who received chemotherapy as second-line treatment were treated with equal probability with paclitaxel or carboplatin. Patients who died were assumed to have received hospice care once before their death.

### 2.2 Model overview

**Figure 1:**
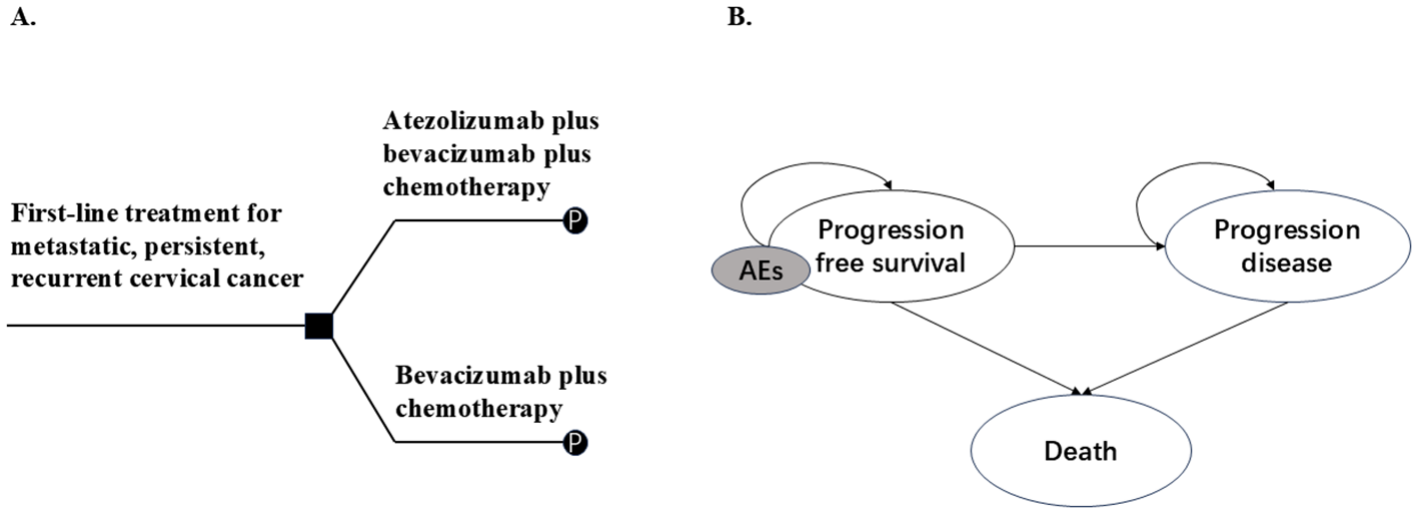
Model overview. **(A)** Decision tree, **(B)** Partitioned survival model. Patients enter the model in a progression-free survival state and may receive atezolizumab plus bevacizumab plus chemotherapy or bevacizumab plus chemotherapy as first-line therapy **(A)**, followed by entry into a partitioned survival model **(B)**, they may remain in the progression-free survival state or enter the progression or death state. Patients may experience AEs during treatment. AEs: Adverse effects as defined by the National Cancer Institute-Common Terminology Criteria for Adverse Events.

Based on the characteristics of the tumor, a PSM was developed by Microsoft Excel^®^ (Microsoft Corp, Redmond, WA, USA) to evaluate the cost-effectiveness of ABC therapy versus BC therapy for women with metastatic, persistent, or recurrent cervical cancer. PSM was composed of three health states: PFS, PD, and death (D). The proportions of patients in different health states at different cycles were obtained from the PFS and OS curves published in the BEATcc clinical trial, as well as parameter fitting and extrapolation. In addition, we considered the reduction in the quality of life of patients and treatment costs resulting from severe adverse events (AEs). To simplify the model, only AEs with an incidence of ≥5% and a severity of ≥ grade 3 were included (**Figure 1**).

From the perspective of US healthcare payers, we evaluated the total cost expenditure and quality-adjusted life year (QALYs) benefits for two arms of patients over a 20-year lifetime horizon (at which point >98% patients were dead). The primary outcome was the incremental cost-effectiveness ratio (ICER); if the ICER was below the per capita willingness-to-pay (WTP) threshold, it indicated that the alternative therapy was cost-effective. Based on the findings of a previous study (14), the WTP for US cancer in the United States was $150,000/QALY. To mitigate the impact of inflation, costs and benefits were discounted at a 3% annual rate.

### 2.3 Clinical data inputs

Based on the published Kaplan-Meier survival curves of the BC and ABC arms in the BEATcc clinical trial, individual patient data were reconstructed using the GetData Graph Digitizer (version 2.26; www.getdata.graph.digitizer.com) and R software (17). The parametric survival analysis method was used to extrapolate the long-term survival status of the patients. Based on the National Institute for Health and Care Excellence (NICE) full list of technical support documents (TSDs) for pharmacoeconomic evaluation and traditional parametric survival analyses (Weibull, Log-normal, Log-logistic, Gamma, Gen-gamma, Gompertz, and Exponent) were used for patient survival fitting and extrapolation (18). However, traditional parametric methods can only capture a specific shape of the risk function and tend to ignore the complexity of the actual risk function (19), NICE published the TSD_21_ document in 2020, which suggests using flexible parametric survival analyses (such as piecewise models and cure models) for long-term patient survival extrapolation (20). In this study, based on traditional parameters, five flexible parameters (Royston-Parmar spline model (RP, harzed, odd, normal, knots = 1,2,3), restricted cubic spline model (knots = 3,4), fractional polynomials (1&2), generalized additive model, and piecewise exponential model) were added to fit and extrapolate the long-term survival of patients. The Akaike, Bayesian, and Watanabe-Akaike information criteria were used to determine the best-fitting distribution from the 12 distributions. The results showed that the log-logistic distribution provided the best OS fit (both BC and ABC arms), whereas RP provided the best PFS fit (odds-2 for BC arm and harzed-3 for ABC arm) (**Supplementary Table S3**). **Supplementary Figure S1** shows the reconstructed, extrapolated survival curves.

### 2.4 Costs and utilities

Only the direct medical costs were considered from the perspective of healthcare providers. As our study was based on the BEATcc clinical trial, the treatment pathway was assumed to be the same as that in the clinical trial. To align the model more closely with the real-world situation, relevant cost items, including fees for injection management, follow-up visits, hospice care, and adverse events (AEs), were considered. The cost data for pharmaceuticals, diagnostic procedures, and follow-up visits were sourced from the Centers for Medicare and Medicaid Services (CMS) database (11, 21). These nationally representative cost estimates reflect average healthcare system expenditures across the U.S., although actual treatment costs may vary based on geographical location, healthcare facility type, insurance coverage, and other factors. The variability of costs was further discussed in the one-way sensitivity and probabilistic sensitivity analyses. For AEs management costs, we searched for a high-level evidence-based rationale to obtain the recommended treatment protocols, and the corresponding cost data from the CMS database, best supportive care, and hospice care costs were derived from previously published literature (15, 22–27). All costs were adjusted to the 2024 values using the U.S. Healthcare Consumer Price Index (28) (**Table 1**).

**Table 1:** Basic model inputs.

Parameter	Base	Min	Max	Distribution	Reference
Clinical data input for the ABC arm					
Royston-Parmar Spline Model _hazard3 PFS survival model	gamma0 = –5.334; gamma1 = 0.995; gamma2 = –2.079; gamma3 = 3.111; gamma4 = –0.902	**-**	**-**	–	Model fitting
Log-logistic OS survival model	Shape:1.89; Scale: 30.33	**-**	**-**	**-**	Model fitting
Clinical data input for the BC arm					
Royston-Parmar Spline Model _ odd2 PFS survival model	gamma0 = –4.754; gamma1 = 1.187; gamma2 = –1.09; gamma3 = 1.278	**-**	**-**	**-**	Model fitting
Log-logistic OS survival model	Shape:2.05; Scale:23.39	**-**	**-**	**-**	Model fitting
Drug costs ($)					
bevacizumab per cycle	8617.84	6894.27	10341.41	Gamma	(11)
atezolizumab per cycle	10072.2	8057.76	12086.64	Gamma	(11)
pembrolizumab per cycle	12186.56	9749.248	14623.872	Gamma	(11)
cemiplimab per cycle	11178	8942.4	13413.6	Gamma	(11)
Carboplatin per cycle	58.09	46.47	69.7	Gamma	(11)
Cisplatin per cycle	31.9	25.5	38.29	Gamma	(11)
paclitaxel per cycle	36.53	29.22	43.83	Gamma	(11)
**Chemotherapy infusion ($)**	186.53	149.22	223.84	Gamma	(11)
Laboratory tests ($)					
Whole blood sample for DNA analysis per time	760	608	912	Gamma	(21)
CT & MRI per time	566.53	453.22	679.84	Gamma	(21)
biomarker analysis per time	20.81	16.65	24.97	Gamma	(21)
routine checkup per time	137.83	110.26	165.4		(21)
**Follow-up visit per cycle ($)**	17.81	14.24	21.37	Gamma	(21)
**Supportive care per cycle ($)**	4824.06	3859.25	5788.87	Gamma	(21)
**Hospice care per event ($)**	11269	9015.2	13522.81	Gamma	(21)
AEs treatment costs ($)					
Peripheral or sensory neuropathy	1139.04	911.23	1366.85	Gamma	(11, 23)
Anemia	1450.5	1160.4	1740.6	Gamma	(11, 22)
Neutropenia/Febrile neutropenia	29643.23	23714.58	35571.87	Gamma	(25, 28)
Thrombocytopenia	2487.64	1990.11	2985.17	Gamma	(26, 28)
Hypertension	2637.46	2109.97	3164.95	Gamma	(24, 28)
Risk of AEs in ABC arm (%)					
Peripheral or sensory neuropathy	7	5.6	8.4	Beta	(9)
Anemia	14	11.2	16.8	Beta	(9)
Neutropenia/Febrile neutropenia	23	18.4	27.6	Beta	(9)
Thrombocytopenia	5	4	6	Beta	(9)
Hypertension	18	14.4	21.6	Beta	(9)
Asthenia	11	8.8	13.2	Beta	(9)
Risk of AEs in BC arm (%)					
Peripheral or sensory neuropathy	4	3.2	4.8	Beta	(9)
Anemia	7	5.6	8.4	Beta	(9)
Neutropenia/Febrile neutropenia	27	21.6	32.4	Beta	(9)
Thrombocytopenia	6	4.8	7.2	Beta	(9)
Hypertension	16	12.8	19.2	Beta	(9)
Asthenia	9	7.2	10.8	Beta	(9)
Health utility					
PFS	0.71	0.568	0.852	Beta	(29)
PD	0.58	0.464	0.696	Beta	(30)
Health disutility					
Peripheral or sensory neuropathy	–0.049	–0.039	–0.059	Beta	(15)
Anemia	0	/	/	/	(31)
Neutropenia/Febrile neutropenia	0	/	/	/	(31)
Thrombocytopenia	0	/	/	/	(31)
Hypertension	–0.03	–0.024	–0.036	Beta	(28)
Asthenia	–0.074	–0.0592	–0.0888	Beta	(28)
**BSA (m^2^)**	1.95	1.56	2.34	Normal	(16)
**Discount rate (%)**	0.03	0	0.08	Fixed	

BC, bevacizumab plus chemotherapy; ABC, atezolizumab plus bevacizumab plus chemotherapy; PFS, progression-free survival; OS, overall survival; PD, progressive disease; D, death; AEs, adverse events; and BSA, body surface area.

The utility values for different survival states of patients with cervical cancer were derived from the studies by Monk (29) and Oaknin (30) et al. Monk et al. published utility values for the baseline states of patients with persistent, metastatic, and recurrent cervical cancer, and Oaknin et al. published utility values for patients with disease progression. The negative utility values for AEs were derived from the published literature (15, 27, 32) and the utility for death was 0 (**Table 1**).

### 2.5 Sensitivity analysis

One-way sensitivity, probabilistic sensitivity, and scenario analyses were conducted to assess the robustness of the models. One-way sensitivity analysis examines the effect of varying a parameter on the model results, whereas other parameters are held constant, either within the 95% CI reported in the literature or within a specific interval (10%–30%). In this study, an evaluation interval of ±20% was used for parameters for which there was no 95% CI (either cost or utility value parameters). Because the prices of the ICIs with the greatest impact on outcomes did not fluctuate by more than 20% during 2014–2021 (atezolizumab 16%, pembrolizumab 14%, and cemiplimab 11%) (33), and the difference in health utility values for patients with cervical cancer across different stages did not exceed 20% as well (34). The results of the one-way sensitivity analysis are shown in a tornado diagram. Probabilistic sensitivity analysis examines how simultaneous variations in all parameters affect the model output. All parameters were randomly changed in pre-specified distributions throughout 1000 iterations of Monte Carlo simulations, with beta distributions used for probability and utility values and gamma distributions used for cost parameters. A scatter plot and acceptable cost-effectiveness curve were plotted to show the results. Owing to the uncertainty associated with curve extrapolation in the scenario analysis, we examined the cost-effectiveness of the two treatment regimens during the follow-up year of the clinical trial (4.5y), as the survival benefit for patients was determined during the 4.5-year observation period. In addition, we examined whether a 10% reduction in the price of atezolizumab impacted the cost-effective of the model.

## 3 Results

### 3.1 Base-case results

Over 20y, patients in the BC arm paid $2762013.43 and acquired 1.67 QALYs, whereas those in the ABC group paid $2878199.88 and acquired 2.27 QALYs. With incremental costs of $116186.4452 and 0.6 incremental QALY, the ABC therapy was associated with an ICER of $193926.48/QALY, which was above the $150,000 WTP threshold, suggesting that ABC therapy was not cost-effective for US healthcare payers (**Table 2**).

**Table 2:** Model-generated results.

Baseline results: Cost-effectiveness analysis (20 y)					
Arm	C ($)	E (QALY)	Incr C	Incr E	ICER($/QALY)
BC	2762013.43	1.67			
ABC	2878199.88	2.27	116186.4452	0.60	193926.48
Scenario analysis 1. Simulated lifetime horizon: 4.5y					
BC	616807.6965	1.16			
ABC	665634.6421	1.45	48826.95	0.29	168482.26
Scenario analysis 2. Cost of atezolizumab reduced by 10%					
BC	2762013.44	1.67			
ABC	2834825.99	2.27	72812.56	0.60	121531.24

BC, bevacizumab plus chemotherapy; ABC, atezolizumab plus bevacizumab plus chemotherapy; C, cost; E, effectiveness; Incr, incremental; and ICER, incremental cost-effectiveness ratio.

### 3.2 Sensitivity analysis

**Figure 2:**
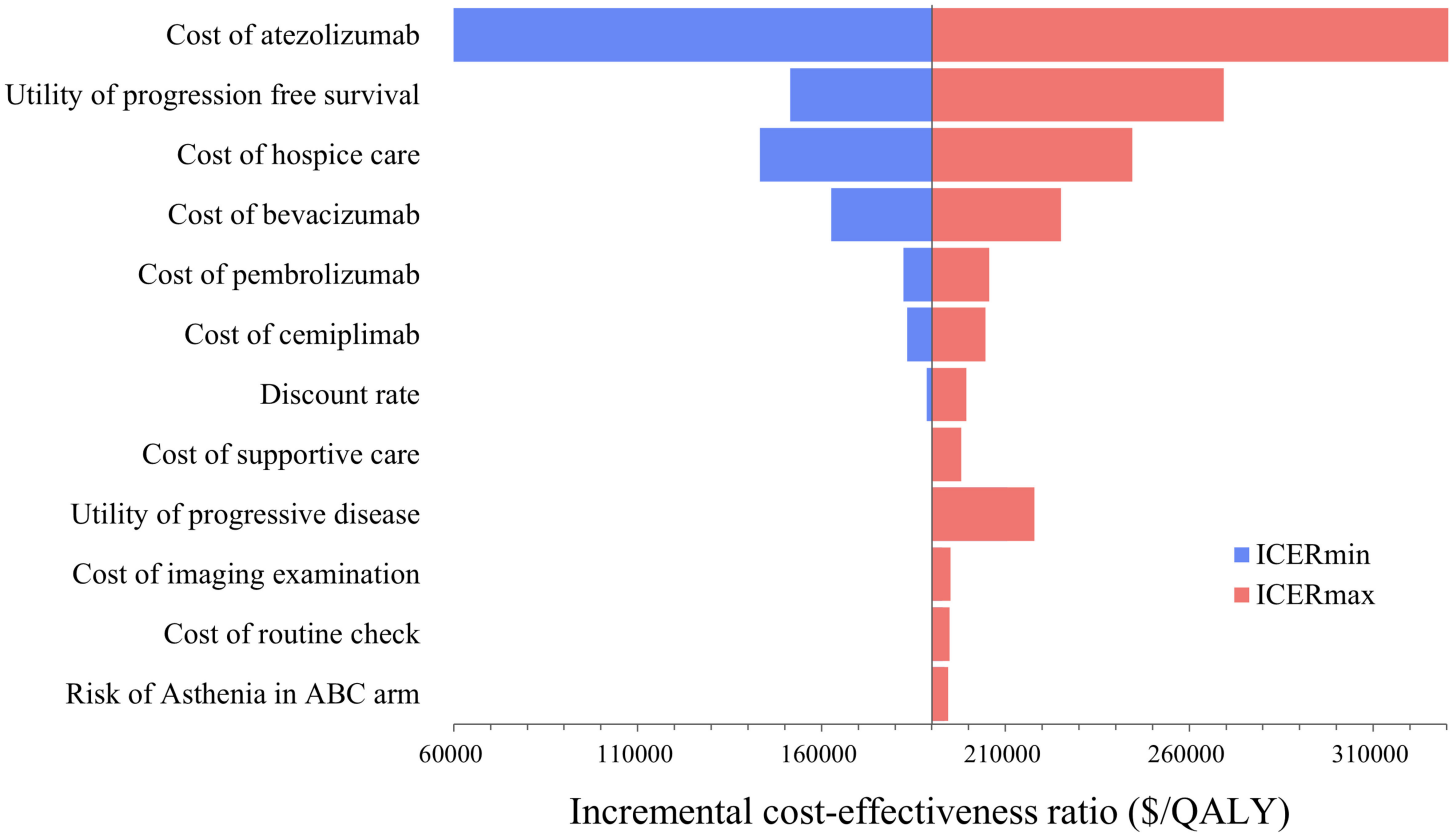
Tornado diagram constructed form the one-way sensitivity analysis.

The tornado diagram (**Figure 2**) of the one-way sensitivity analysis shows that the price of atezolizumab, utility value of the PFS, and cost of hospice care had the greatest effect on the cost-effectiveness of the model. Notably, ABC changed from not cost effective to cost effective when the price of atezolizumab and the cost of hospice care decreased by 20%. Changes in the other factors had no effect on the model outcome (whether it was cost-effective).

**Figure 3:**
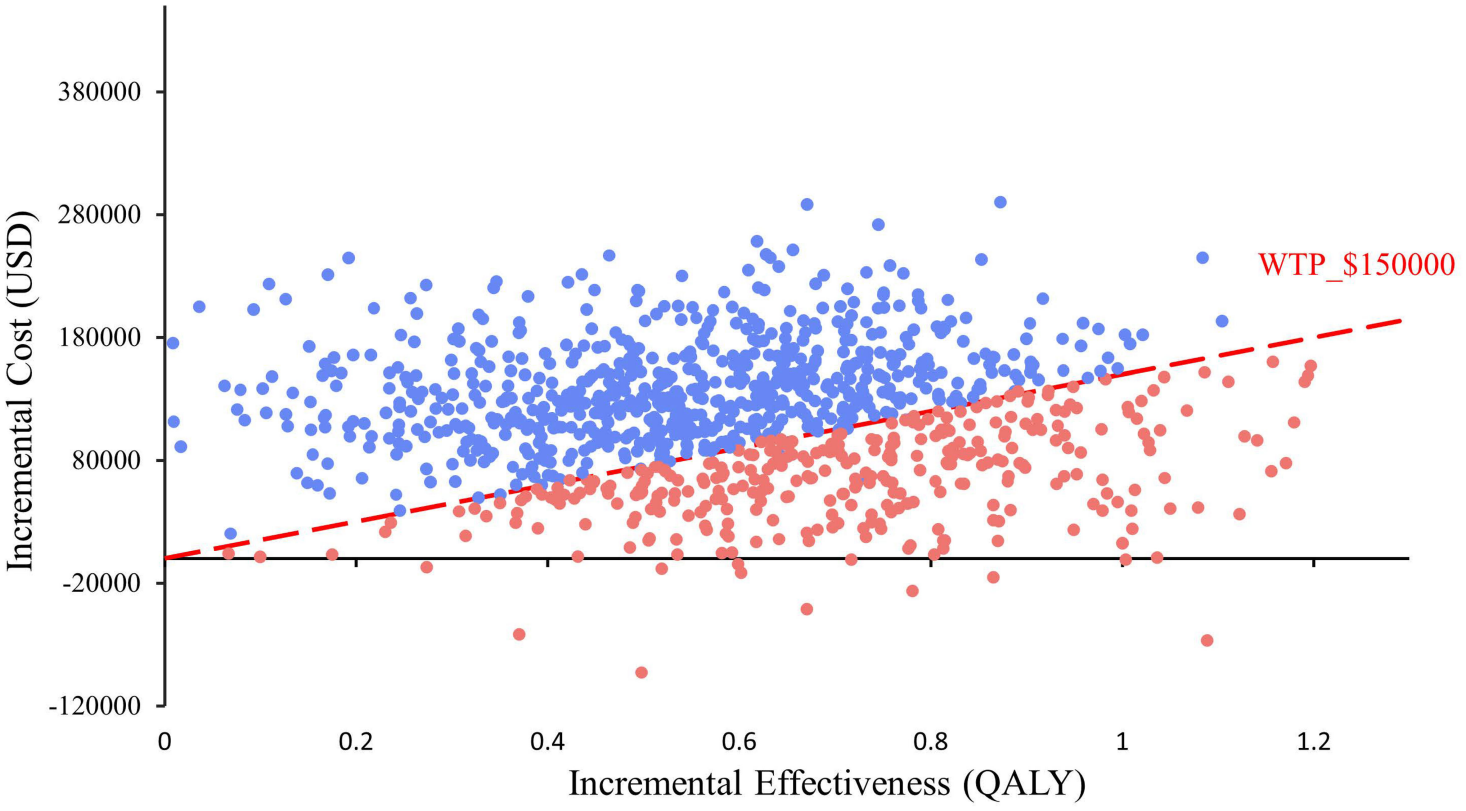
Scatter plot constructed from the probabilistic sensitivity analysis.

**Figure 4:**
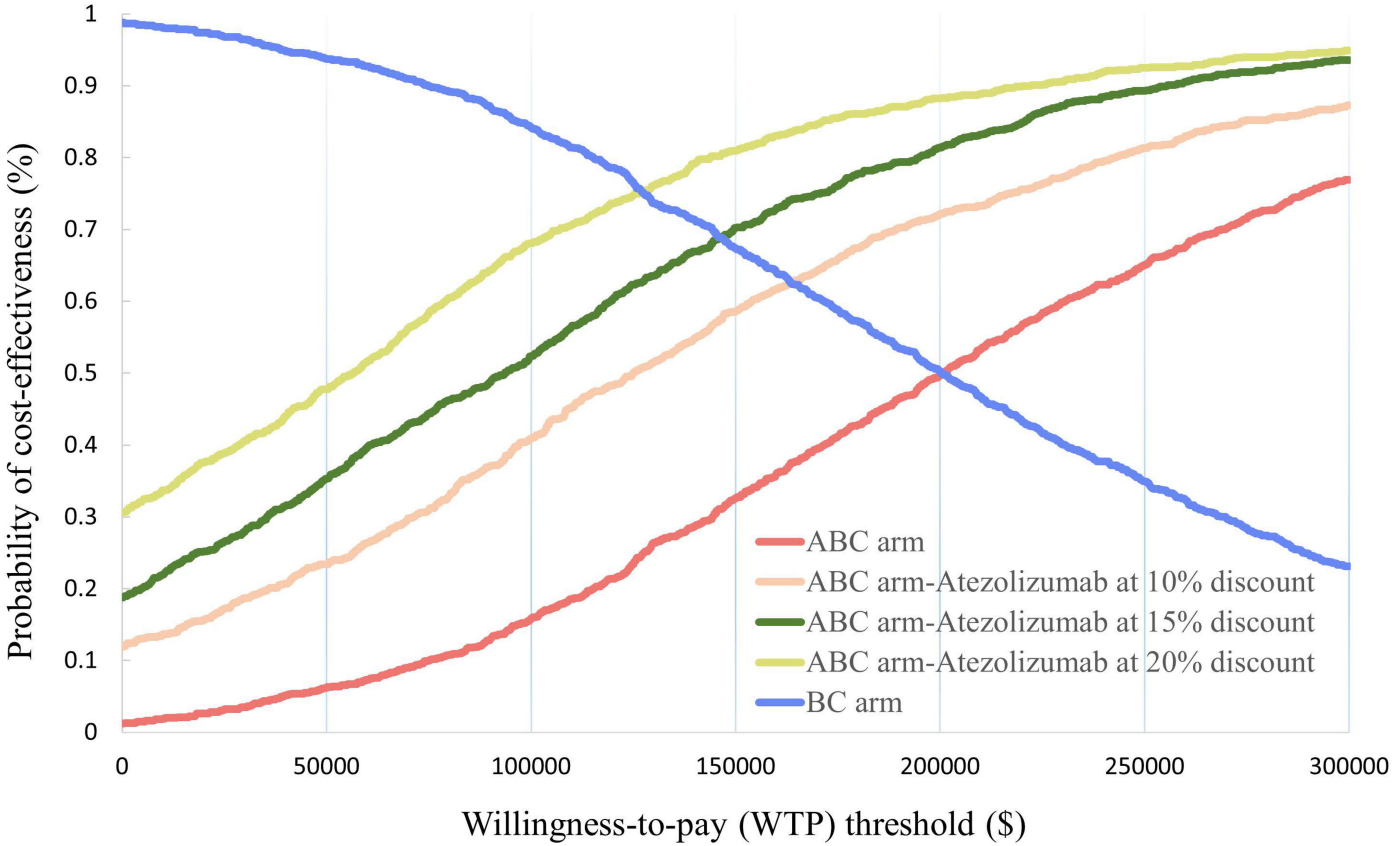
Acceptable cost-effectiveness curve constructed from the probabilistic sensitivity analysis.

In the probabilistic sensitivity analysis, 1000 Monte Carlo simulations showed that at the $150,000/QALY WTP threshold, the probability of ABC therapy being cost-effective was 32.6% (**Figure 3**). When the cost of atezolizumab decreased by 10%, 15%, and 20%, the probability of ABC therapy being cost-effective increased to 58.6%, 70.2%, and 81%, respectively (**Figure 4**).

In the scenario analyses, ABC therapy was continuously less cost-effective than BC therapy at 4.5 y of lifetime, with ICER values of $ 168482.263/QALY. However, when the cost of atezolizumab was reduced by 10%, ABC therapy was more cost effective than BC therapy (**Table 2**).

## 4 Discussion

Compared to other highest-income countries such as England, Canada, and Germany, the US spent approximately twice the amount on healthcare; however, healthcare was less equitably accessible and population health indicators were poorer in the US (35). As novel anticancer drugs became available on the market and healthcare expenditures continued to grow, all of these factors made it more urgent in the US than in other countries to promote health equity and value-based healthcare payments (36). Since 2010, the US has passed the Affordable Care Act (ACA) and the Medicare Access and Children’s Health Insurance Reauthoriation Act (CHIRA), while authorizing the creation of the Center for Medicare and Medicaid Innovation (CMMI) (37, 38), aiming to explore new Medicare payment methods to ensure quality of care while minimizing Medicare spending. In the field of cancer, the first large-scale value-based oncology bundled payment, the Oncology Care Model (OCM), was piloted in 2017. However, studies have shown that the OCM failed to accurately adjust for exponential growth in drug prices, especially for novel therapies (cell and gene therapy) in gynecological oncology (39, 40). Public spending on medicines continues to increase, and health authorities are continuously searching for methods to improve public access to innovative medicines and healthcare affordability, while supporting the development of innovative medicines (41). Economic evaluation based on the decision-analytic model provides an evidence framework that brings together evidence collected from clinical, resource use, and randomized trials for decision making in the healthcare system (42). Notably, PSM and state transition models are the most commonly used model structures for oncology drugs (43).

In terms of the cost-effectiveness of innovative drugs for the first-line treatment of persistent, metastatic, and recurrent cervical cancer, several studies have shown that even if novel medications can significantly improve patient survival, they were still less likely to be cost-effective when compared to that of traditional therapies. Phippen et al. (24) and Minion et al. (44) constructed a simple decision tree model and a Markov model to evaluate the cost-effectiveness of bevacizumab plus chemotherapy versus chemotherapy based on the GOG clinical trial, and the results of both models showed that the combination regimen was not cost-effective (ICER were $155,148/QALY and $252,996,4/QALY, respectively). With the emergence of the Keynote-826 clinical trial, pembrolizumab became a novel first-line treatment option for patients with advanced cervical cancer; Shi et al. (15) constructed a PSM to assess the economics of pembrolizumab plus chemotherapy from a 30-year perspective, which was not cost-effective (ICER was $247663/QALY). Recently, growing evidence has indicated that the inhibition of both angiogenesis and immunosuppression, in combination with chemotherapy, may result in improved and more durable clinical benefits. BEATcc clinical trial (10) showed that, subject to acceptable security, ABC triple therapy significantly improved median PFS and median OS in patients with advanced cervical cancer compared to BC therapy (median PFS 13.7 mo vs. 10.4 mo, median OS 32.1 mo vs. 22.8 mo). Although recent studies have been published on the cost-effectiveness of atezolizumab in the treatment of advanced cervical cancer, our study made optimized choices in terms of time horizon, survival curve extrapolation methodology, and parameter settings which we considered to be more closely aligned with the cost-effectiveness of real-world treatments for patients with metastatic, persistent, and recurrent cervical cancer.

In the present study, PSM was performed to evaluate the cost-effectiveness of adding atezolizumab to standard therapy as a novel first-line option for metastatic, persistent, or recurrent cervical cancer. Cost data were obtained from the Centers for Medicare and Medicaid Services (USA) and the published literature. Efficacy outcomes and utility values were obtained from the BEATcc clinical trial (9) and published literature. Our modeling results showed that the ICER of adding atezolizumab to standard BC therapy was higher than that of the pre-established WTP, both at 20 y and the clinical trial follow-up years ($193,926.48/QALY, $168,482.26/QALY vs. $150,000/QALY). Our findings were consistent with prior observations by Barrington et al. (14), who noted that patients receiving pembrolizumab + BC paid an extra $341,316 per QALY gained, which was much higher than the WTP and was not cost-effective. Notably, the ICER value of ABC therapy was much lower than that of triple therapy containing pembrolizumab ($193,926.48/QALY vs. $341,316/QALY). The one-way sensitivity analysis showed that when the price of atezolizumab was reduced by 20%, ABC therapy changed from being not cost-effective to cost-effective; probabilistic sensitivity analyses showed that ABC had a 32.6% probability of being cost-effective and that percentage increased to 58.6%, 70.2%, and 81% when the price of atezolizumab was reduced by 10%, 15%, and 20%, respectively. Overall, we believe that adding atezolizumab to standard BC therapy can provide a significant survival benefit to patients and can potentially be cost-effective if its price could be marginally reduced. Our study constructed a complete decision analysis framework, including study perspectives, study timeframes, study populations, study methods, interventions, study hypotheses, and discounting, incorporating evidence from all aspects of healthcare to comprehensively evaluate the cost-effectiveness of alternative treatments, with the aim of providing decision-making recommendations for healthcare payers. In terms of model selection, instead of calculating the probability of metastasis between disease states, as state transition models require, we used PSM, which reconstructs patient survival data from survival curves published in clinical trials to determine the proportion of patients in different disease states at each cycle. This enabled more precise modeling of illness states by avoiding the effects of assumptions such as natural mortality and memory loss on the uncertainty of model outcomes. In terms of lifetime horizon, a 20-year time horizon was conducted. Since we aimed to examine the cost-effectiveness of treatment with ABC or BC over the full lifespan of patients with metastatic, persistent, and recurrent cervical cancer, and as stage I and II patients accounted for 45.6% of the total study population in the BEATcc clinical trial, publicly available data show that the 10-year relative survival rates for localized, regional, and distant cervical cancer patients were 88.1%, 51.9% and 14%, respectively (45), the results of a long-term retrospective cohort study from China also showed that the 20-year survival rate for patients with recurrent cervical cancer was approximately 10% (46); thus, we inferred that the survival prognosis for cervical cancer patients was better. Therefore, a 20-year time horizon was used. Notably, when the model simulated to the 20th year, more than 98% of the patients entered the death state, which is in line with the basic statistical inference. Moreover, considering the uncertainty associated with the parametric extrapolation of survival probabilities, we also accounted for the cost-effectiveness of the two treatment regimens during the 4.5 y of clinical trial follow-up. A half-period correction was applied to remove the discretization bias due to the difference in the model period (3 wk/period) and survival curve period (1 mo/period), and a series of sensitivity analyses were performed to validate model stability. In terms of methods, five flexible survival curve extrapolation methods were added to the traditional seven-parameter fitting methods, and the results showed that RP was better fitted to patients in PFS. Finally, we diligently considered real-world consultation costs and magnitude of change, and considering patient survival utility values, we used specific survival utility values for patients with recurrent, persistent, and metastatic cervical cancers (29, 30). This provides a significant advantage compared with previous studies (14, 15, 31, 47). Subgroup analyses were not performed because the clinical trial enrolled an all-comer population without biomarker selection.

Our study has some limitations. First, our study was based on outcomes from clinical trials, with strict inclusion and exclusion criteria and established diagnostic procedures that made it difficult to reflect the efficacy of a drug when used in real-world patients. Several studies have shown that the efficacy of drugs in the real world was inferior to that in clinical trials (48, 49). This is a common problem in health economic assessments based on clinical trials, which is expected to be resolved as real-world research expands and an economic evaluation system based on real-world outcomes can be established. Second, our study did not include subgroup analyses of patients with specific cancer characteristics because the efficacy of patients with different PD-1 expression statuses was not addressed in the clinical trials. However, PD-1 expression levels may affect patient outcomes and thus cost-effectiveness results, and further analyses of this component will hopefully be conducted as the clinical trials continue. Third, only grade ≥3 AEs with an incidence of ≥5% were considered and the failure to include all AEs may have led to an underestimation of the cost and an overestimation of the survival benefit. In addition, to simplify the model, we assumed that patients with PD were treated with chemotherapy (paclitaxel or carboplatin), ICIs (pembrolizumab or cemiplimab), or best supportive care, whereas the selection of real-world second-line treatment is much more complex. Sensitivity analyses were conducted to verify the stability of the model for the above issues, and the results indicated that the uncertainty parameters did not affect the results of the model.

Finally, atezolizumab has been approved for the treatment of a variety of cancers, including non-small-cell lung cancer, endometrial cancer, uroepithelial cancer, and hepatocellular carcinoma, and its cost-effectiveness for different cancers has been widely evaluated, but almost all studies have shown that atezolizumab-containing treatment regimens are not cost-effective (50–53), which suggests that atezolizumab may be overpriced. Our study also demonstrated that atezolizumab-containing regimens (ABC) were not cost-effective compared to conventional first-line regimens; however, it is noteworthy that with a WTP of $150,000, the likelihood of ABC being cost-effective increased to 58.6% when the price of atezolizumab was reduced by only 10% (ICER = $121,531.24/QALY). This suggests that drug prices should be appropriately lowered to improve the cost-effectiveness and reduce financial pressure on patients and health insurance payers.

## 5 Conclusion

In summary, from the perspective of US healthcare payers, the addition of atezolizumab to the first-line standard therapy for patients with metastatic, persistent, and recurrent cervical cancer was not cost-effective, although it may provide a survival benefit to patients. The price of atezolizumab is a key element influencing the cost-effectiveness of ABC therapy, and an appropriate price decrease (10%) is recommended for atezolizumab to improve the cost-effectiveness of ABC therapies.

## Data Availability

The original contributions presented in the study are included in the article/**Supplementary Material**. Further inquiries can be directed to the corresponding author.
