# Supplementary material for: Atezolizumab plus bevacizumab and chemotherapy as first-line therapy for cervical cancer: a cost-effectiveness analysis in the US
Source: Front Immunol. 2024 Nov 27;15:1481584. doi: 10.3389/fimmu.2024.1481584 (PMC11631890; doi:10.3389/fimmu.2024.1481584)

Supplementary Material

Atezolizumab plus bevacizumab and chemotherapy as first-line therapy for cervical cancer: a cost-effectiveness analysis in the US

Yingtao Lin, Cijuan Li, Chang Wang, Jian Chen, Yuanqing Huang

*** Correspondence:** Yuanqing Huang: 383196814@qq.com

# Table S1. CHEERS Checklist.

# Table S2. Summary of patient baseline information.

# Table S3. Summary of statistical goodness-of-fit of KM curves in BEATcc clinical trial.

# Figure S1. The rebuilt extrapolated survival curves for BC arm and ABC arm.

# Table S1. CHEERS Checklist.

| **Section/topic** | **Item No** | **Guidance for reporting** | **Reported in Page No.** |
| --- | --- | --- | --- |
| **Title** | | |  |
| Title | 1 | Identify the study as an economic evaluation and specify the interventions being compared. | 1 |
| **Abstract** | | |  |
| Abstract | 2 | Provide a structured summary that highlights context, key methods, results, and alternative analyses. | 1, 2 |
| **Introduction** | | |  |
| Background and objectives | 3 | Give the context for the study, the study question, and its practical relevance for decision making in policy or practice. | 2, 3 |
| **Methods** | | |  |
| Health economic analysis plan | 4 | Indicate whether a health economic analysis plan was developed and where available. | 3,4 |
| Study population | 5 | Describe characteristics of the study population (such as age range, demographics, socioeconomic, or clinical characteristics). | 3 |
| Setting and location | 6 | Provide relevant contextual information that may influence findings. | 4,5 |
| Comparators | 7 | Describe the interventions or strategies being compared and why chosen. | 2,3 |
| Perspective | 8 | State the perspective(s) adopted by the study and why chosen. | 3,4 |
| Time horizon | 9 | State the time horizon for the study and why appropriate. | 4 |
| Discount rate | 10 | Report the discount rate(s) and reason chosen. | 4 |
| Selection of outcomes | 11 | Describe what outcomes were used as the measure(s) of benefit(s) and harm(s). | 4 |
| Measurement of outcomes | 12 | Describe how outcomes used to capture benefit(s) and harm(s) were measured. | 4 |
| Valuation of outcomes | 13 | Describe the population and methods used to measure and value outcomes. | 3 |
| Measurement and valuation of resources and costs | 14 | Describe how costs were valued. | 4,5 |
| Currency, price date, and conversion | 15 | Report the dates of the estimated resource quantities and unit costs, plus the currency and year of conversion. | 5 |
| Rationale and description of model | 16 | If modelling is used, describe in detail and why used. Report if the model is publicly available and where it can be accessed. | 4 |
| Analytics and assumptions | 17 | Describe any methods for analysing or statistically transforming data, any extrapolation methods, and approaches for validating any model used. | 4 |
| Characterizing heterogeneity | 18 | Describe any methods used for estimating how the results of the study vary for subgroups. | Not applicable |
| Characterizing distributional effects | 19 | Describe how impacts are distributed across different individuals or adjustments made to reflect priority populations. | Not applicable |
| Characterizing uncertainty | 20 | Describe methods to characterise any sources of uncertainty in the analysis. | 5 |
| Approach to engagement with patients and others affected by the study | 21 | Describe any approaches to engage patients or service recipients, the general public, communities, or stakeholders (such as clinicians or payers) in the design of the study. | Not applicable |
| **Results** | | |  |
| Study parameters | 22 | Report all analytic inputs (such as values, ranges, references) including uncertainty or distributional assumptions. | 4,5, Table 1. Basic model inputs. |
| Summary of main results | 23 | Report the mean values for the main categories of costs and outcomes of interest and summarise them in the most appropriate overall measure. | 5, Table 2. Model generated results |
| Effect of uncertainty | 24 | Describe how uncertainty about analytic judgments, inputs, or projections affect findings. Report the effect of choice of discount rate and time horizon, if applicable. | 6, Figure 2: tornado diagram, Figure 3: scatter plot and an acceptable cost-effectiveness curve, Figure 4: acceptable cost-effectiveness curve, Table 2. Model generated results |
| Effect of engagement with patients and others affected by the study | 25 | Report on any difference patient/service recipient, general public, community, or stakeholder involvement made to the approach or findings of the study | Not applicable |
| **Discussion** | | |  |
| Study findings, limitations, generalizability, and current knowledge | 26 | Report key findings, limitations, ethical or equity considerations not captured, and how these could affect patients, policy, or practice. | 8,9 |
| **Other relevant information** | | | |
| Source of funding | 27 | Describe how the study was funded and any role of the funder in the identification, design, conduct, and reporting of the analysis | 14 |
| Conflicts of interest | 28 | Report authors conflicts of interest according to journal or International Committee of Medical Journal Editors requirements. | 14 |

# *Reference: Husereau D, Drummond M, Augustovski F, et al. Consolidated Health Economic Evaluation Reporting Standards 2022 (CHEERS 2022) Explanation and Elaboration: A report of the ISPOR CHEERS II Good Practices Task Force. Value Health. 2022;25(1):10-31.*

# Table S2. Summary of patient baseline information.

| **Characteristic** | **atezolizumab plus bevacizumab plus chemotherapy (ABC arm; n=206)** | **bevacizumab plus chemotherapy (BC arm; n=204)** |
| --- | --- | --- |
| **Age, years** | 51.0 (43.0-60.0) | 52.5 (43.5-61.0) |
| ＜65 years | 171 (83%) | 168 (82%) |
| ≥65 years | 35 (17%) | 36 (18%) |
| **Gynecologic Oncology Group or Eastern Cooperative Oncology Group performance status** | | |
| 0 | 138 (67%) | 128 (63%) |
| 1 | 68 (33%) | 73 (36%) |
| Missing data | 0 | 3 (1%) |
| **Race or ethnicity** | | |
| White | 111 (54%) | 113 (55%) |
| Asian | 31 (51%) | 27 (13%) |
| Latin | 8 (4%) | 10 (5%) |
| Arab | 2 (1%) | 3 (1%) |
| Black | 3 (1%) | 2 (1%) |
| Gypsy | 1(<1%) | 0 |
| Not available | 50 (24%) | 49 (24%) |
| **Geographical region** | | |
| Europe | 166 (81%) | 173 (85%) |
| Japan | 30 (15%) | 26 (13%) |
| USA | 10 (5%) | 5 (2%) |
| **Histological subtype** | | |
| Squamous cell carcinoma | 164 (80%) | 157 (77%) |
| Adenocarcinoma | 36 (17%) | 43 (21%) |
| Adenosquamous cell carcinoma | 6 (3%) | 4 (2%) |
| **International Federation of Gynecology and Obstetrics stage at diagnosis** | | |
| I | 31 (15%) | 42 (21%) |
| II | 61 (30%) | 53 (26%) |
| III not otherwise specified | 14 (7%) | 12 (6%) |
| IIIA | 2 (1%) | 4 (2%) |
| IIIB | 34 (17%) | 22 (11%) |
| IV not otherwise specified | 5 (2%) | 8 (4%) |
| IVA | 11 (5%) | 6 (3%) |
| IVB | 43 (21%) | 50 (25%) |
| Not assessed or pre-invasive | 4 (2%) | 5 (2%) |
| Missing | 1 (<1%) | 2 (1%) |
| **Disease status at screening** | | |
| Newly diagnosed metastatic disease (stage IVB) | 43 (21%) | 47 (23%) |
| Recurrent (includes patients with distant disease) | 150 (73%) | 151 (74%) |
| Persistent | 13 (6%) | 6 (3%) |
| **Disease location at screening** | | |
| Pelvic and distant | 102 (50%) | 90 (44%) |
| Distant only | 71 (34%) | 74 (36%) |
| Pelvic only | 33 (16%) | 40 (20%) |
| **Initial therapy** | | |
| Concurrent chemoradiotherapy | 70 (34%) | 85 (42%) |
| Surgery | 9 (4%) | 14 (7%) |
| Surgery followed by chemoradiotherapy | 64 (31%) | 44 (22%) |
| Surgery followed by radiotherapy | 5 (2%) | 11 (5%) |
| Primary radiotherapy | 2 (1%) | 3 (1%) |
| None | 56 (27%) | 47 (23%) |
| **Investigator-selected platinum backbone** | | |
| Cisplatin | 124 (60%) | 119 (58%) |
| Carboplatin | 82 (40%) | 85 (42%) |

# Table S3. Summary of statistical goodness-of-fit of KM curves in BEATcc clinical trial.

| Table S3 Summary of statistical goodness-of-fit of KM curves in BEATcc clinical trial. | | | | | | | | |
| --- | --- | --- | --- | --- | --- | --- | --- | --- |
| **Models** | **PFS curve** | | | | **OS curve** | | | |
|  | **AIC** | **BIC** | **WAIC** | **Other information** | **AIC** | **BIC** | **WAIC** | **Other information** |
| **ABC arm,** | | | | | | | | |
| **Standard parametric models for survival analysis** | | | | | | | | |
| exp | 1165.4 | 1168.7 | 1165.5 | Rate = 0.04 | 1000.4 | 1003.7 | 1000.1 | Rate = 0.02 |
| weibull | 1160.8 | 1167.4 | 1160.6 | shape = 1.21; scale = 0.02 | 979.4 | 986.0 | 979.1 | shape =1.57; scale = 0.003 |
| lnorm | 1140.2 | 1146.8 | 1140.9 | Meanlog = 2.8; sdlog = 1.0 | 987.3 | 993.9 | 989.3 | Meanlog = 3.44 ; sdlog = 0.9998 |
| llog | 1139.2 | 1145.9 | 1139.4 | shape = 1.7; scale = 15.95 | **978.5** | **985.2** | **978.9** | **shape =1.89; scale = 30.33** |
| gam | 1155.9 | 1162.5 | 1155.9 | shape = 1.46; rate = 0.06 | 978.9 | 985.5 | 979.2 | shape =1.90; rate =0.05 |
| ggam | 1142.1 | 1152.1 | 1147.2 | mu = 2.78; sigma = 1.03; Q = -0.06 | 980.9 | 990.9 | 981.1 | mu = 3.61; sigma =0.71; Q = 0.77 |
| gomp | 1167.3 | 1174.0 | 1167.3 | shape = -0.002; rate = 0.04 | 986.6 | 993.3 | 986.5 | shape = 0.04; rate = 0.01 |
| **Flexible methods for survival analysis** | | | | | | | | |
| rp_ hazard (knots = 1) | **1121.8** | **1138.4** | **1126.9** | **gamma0 = -5.334; gamma1 = 0.995; gamma2 = -2.079; gamma3 = 3.111; gamma4 = -0.902** | 983.4 | 1000.1 | 981.9 | gamma0 = -5.586; gamma1 = 1.103; gamma2 = -0.415; gamma3 = 0.388; gamma4 = 0.274 |
| rp _ hazard (knots = 2) | 1125.5 | 1138.8 |  | gamma0 = -5.6474; gamma1 = 2.2353; gamma2 = -0.6687; gamma3 = 0.9907 | 981.5 | 994.83 |  | gamma0 = -5.634; gamma1 = 1.225; gamma2 = -0.555; gamma3 = 0.845 |
| rp _ hazard (knots = 3) | 1128.8 | 1138.8 |  | gamma0 = -7.1758; gamma1 = 4.1429; gamma2 = 0.2469 | 981.2 | 991.13 |  | gamma0 = -6.1449; gamma1 = 1.8566; gamma2 = 0.0357 |
| rp _ odd (knots = 1) | 1121.6 | 1138.3 |  | gamma0 = -5.408; gamma1 = 0.88; gamma2 = -2.009; gamma3 = 2.814; gamma4 = -0.659 | 983.4 | 1000.1 |  | gamma0 = -5.5994; gamma1 = 1.0519; gamma2 = -0.2946; gamma3 = 0.3113; gamma4 = 0.0358 |
| rp _ odd (knots = 2) | 1123.2 | 1136.5 |  | gamma0 = -5.4722; gamma1 = 1.6915; gamma2 = -1.0268; gamma3 = 1.3714 | 981.5 | 994.8 |  | gamma0 = -5.606; gamma1 = 1.119; gamma2 = -0.375; gamma3 = 0.486 |
| rp_ odd (knots = 3) | 1129.0 | 1139.0 |  | gamma0 = -7.0651; gamma1 = 3.8599; gamma2 = 0.1905 | 978.0 | 990.0 |  | gamma0 = -5.8244; gamma1 = 1.4289; gamma2 = -0.0601 |
| rp_ normal (knots = 1) | 1122.0 | 1138.6 |  | gamma0 = -2.778; gamma1 = 0.229; gamma2 = -1.04; gamma3 =1.354; gamma4 = -0.236 | 983.0 | 999.6 |  | gamma0 = -2.7177; gamma1 = 0.2624; gamma2 = -0.0932; gamma3 = -0.1135; gamma4 = 0.2335 |
| rp_ normal (knots = 2) | 1122.3 | 1135.6 |  | gamma0 = -2.646; gamma1 = 0.328; gamma2 = -0.823; gamma3 = 1.026 | 981.0 | 994.3 |  | gamma0 = -2.716; gamma1 = 0.267; gamma2 = -0.221; gamma3 = 0.227 |
| rp_ normal (knots = 3) | 1141.55 | 1151.5 |  | gamma0 = -2.9046; gamma1 = 1.1420; gamma2 = 0.0152 | 979.6 | 989.6 |  | gamma0 = -2.7106; gamma1 = 0.3375; gamma2 = -0.0978 |
| rcs (knots = 3) | 1121.8 | 1138.4 |  | gamma0 = -5.334; gamma1 = 0.995; gamma2 = -2.079; gamma3 = 3.111; gamma4 = -0.902 | 983.4 | 1000.1 |  | gamma0 = -5.586; gamma1 = 1.103; gamma2 = -0.415; gamma3 = 0.388; gamma4 = 0.274 |
| rcs (knots = 4) | 1123.7 | 1143.7 |  | gamma0 = -5.401; gamma1 = 0.879; gamma2 = -2.345; gamma3 = 2.995; gamma4 = -0.118; gamma5 = -0.398 | 985.0 | 1005.0 |  | gamma0 = -5.559; gamma1 = 1.045; gamma2 = -0.190; gamma3 = -1.279; gamma4 = 2.968; gamma5 = -1.535 |
| fp1 | 1128.8 | 1138.8 |  | gamma0 = -7.1758; gamma1 = 4.1429; gamma2 = 0.2469 | 981.2 | 991.1 |  | gamma0 = -6.1449; gamma1 = 1.8566; gamma2 = 0.0357 |
| fp2 | 1125.5 | 1138.8 |  | gamma0 = -5.6474; gamma1 = 2.2353; gamma2= -0.6687; gamma2 = 0.9907 | 981.5 | 994.8 |  | gamma0 = -5.634; gamma1 = 1.225; gamma2 = -0.555; gamma2 = 0.845 |
| gam | 1126.3 | 1140.5 |  | Intercept = -1.13; s(time).1 = 1.7857; s(time).2 = 1.6284; s(time).3 = 3.4442; s(time).4 = 1.8288; k = 5 | 986.6 | 999.3 |  | Intercept = -1.4568; s(time).1 = 1.3229; s(time).2 = 1.8548; s(time).3 = 2.6534; s(time).4 = 1.9393; k = 5 |
| pem |  |  | 1145.8 | Change point1 = 5.157; Change point2 = 16.485;  lambda1 = 0.0166; lambda2 = 0.0679; lambda3 = 0.0253 |  |  | 982.6 | Change point1 = 7.992;  lambda1 = 0.00847; lambda2 = 0.02785 |
| **BC arm** | | | | | | | | |
| exp | 1261.6 | 1264.9 | 1261.5 | Rate = 0.06 | 1158.7 | 1162.0 | 1158.4 | Rate = 0.03 |
| weibull | 1240.7 | 1247.3 | 1240.6 | shape = 1.36; scale = 0.02 | 1129.7 | 1136.4 | 1129.7 | shape = 1.57; scale =0.0045 |
| lnorm | 1225.0 | 1231.7 | 1226.2 | Meanlog = 2.41; sdlog = 0.85 | 1128.7 | 1135.3 | 1129.1 | Meanlog = 3.14; sdlog = 0.87 |
| llog | 1214.7 | 1221.3 | 1214.8 | shape = 2.14; scale = 11.08 | **1125.9** | **1132.5** | **1125.9** | **shape =2.05; scale = 23.39** |
| gam | 1231.9 | 1238.5 | 1232.3 | shape = 1.83; rate = 0.12 | 1127.0 | 1133.6 | 1126.9 | shape =2.04; rate = 0.07 |
| ggam | 1225.3 | 1235.3 | 1224.4 | mu = 2.50; sigma = 0.82; Q = 0.22 | 1128.1 | 1138.1 | 1127.3 | mu =3.28; sigma = 0.77; Q = 0.45 |
| gomp | 1259.7 | 1266.3 | 1259.6 | shape = 0.017; rate =0.053 | 1143.5 | 1150.2 | 1143.5 | shape =0.03; rate =0.018 |
| Flexible methods for survival analysis | | | | | | | | |
| rp_ hazard (knots = 1) | 1212.6 | 1229.2 |  | gamma0 = -4.74; gamma1 = 1.32; gamma2 = -0.98; gamma3 = 1.17; gamma4 = -0.008 | 1131.9 | 1148.5 |  | gamma0 = -6.186; gamma1 = 1.87; gamma2 = -0.679; gamma3 = 1.31; gamma4 = -0.602 |
| rp _ hazard (knots = 2) | 1210.6 | 1223.9 |  | gamma0 = -4.756; gamma1 = 1.58; gamma2 = -0.858; gamma3 = 1.092 | 1130.3 | 1143.6 |  | gamma0 = -6.367; gamma1 = 2.033; gamma2 = -0.194; gamma3 = 0.409 |
| rp _ hazard (knots = 3) | 1219.2 | 1229.1 |  | gamma0 = -6.0547; gamma1 = 3.2758; gamma2 = 0.1711 | 1128.6 | 1138.5 |  | gamma0 = -6.6864; gamma1 = 2.2505; gamma2 = 0.1334 |
| rp _ odd (knots = 1) | 1211.8 | 1228.4 |  | gamma0 = -4.7625; gamma1 = 1.2912; gamma2 = -0.2055; gamma3 = -1.2155; gamma4 = 1.6650 | 1131.2 | 1147.8 |  | gamma0 = -6.157; gamma1 = 1.831; gamma2 = -0.721; gamma3 = 1.927; gamma4 = -1.634 |
| rp _ odd (knots = 2) | **1209.6** | **1222.9** | **1210.5** | **gamma0 = -4.754; gamma1 = 1.187; gamma2 = -1.09; gamma3 = 1.278** | 1129.8 | 1143.1 | 1128.5 | gamma0 = -6.3242; gamma1 = 1.9921; gamma2 = 0.0745; gamma3 = -0.1295 |
| rp_ odd (knots = 3) | 1216.3 | 1226.3 |  | gamma0 = -5.481; gamma1 = 2.4415; gamma2 = 0.029 | 1127.8 | 1137.8 |  | gamma0 = -6.2684; gamma1 = 1.9477; gamma2 = -0.0211 |
| rp_ normal (knots = 1) | 1211.8 | 1228.4 |  | gamma0 = -2.4434; gamma1 = 0.3883; gamma2 = -0.111; gamma3 = -1.028; gamma4 = 1.2997 | 1131.3 | 1147.9 |  | gamma0 = -2.985; gamma1 = 0.734; gamma2 = -0.386; gamma3 = 0.800; gamma4 = -0.625 |
| rp_ normal (knots = 2) | 1209.7 | 1223.0 |  | gamma0 = -2.463; gamma1 = 0.3424; gamma2 = -0.7866; gamma3 = 0.8984 | 1129.6 | 1142.9 |  | gamma0 = -3.033; gamma1 = 0.7865; gamma2 = -0.1208; gamma3 = 0.0739 |
| rp_ normal (knots = 3) | 1224.9 | 1234.9 |  | gamma0 = -2.59; gamma1 = 0.8953; gamma2 = -0.031 | 1127.7 | 1137.7 |  | gamma0 = -3.0837; gamma1 = 0.8293; gamma2 = -0.0763 |
| rcs (knots = 3) | 1212.6 | 1229.2 |  | gamma0 = -4.7398; gamma1 = 1.32453; gamma2 = -0.9821; gamma3 = 1.1738; gamma4 = -0.0085 | 1131.9 | 1148.5 |  | gamma0 = -6.186; gamma1 = 1.870; gamma2 = -0.679; gamma3 = 1.310; gamma4 =-0.602 |
| rcs (knots = 4) | 1210.4 | 1230.3 |  | gamma0 = -4.765; gamma1 = 1.6056; gamma2 = 1.1819; gamma3 = -8.4105; gamma4 =10.0428; gamma5 = -2.7179 | 1133.7 | 1153.6 |  | gamma0 =-6.345; gamma1 = 2.023; gamma2 = 0.366; gamma3 = -2.247; gamma4 = 3.403; gamma5 = -1.762 |
| fp1 | 1219.2 | 1229.1 |  | gamma0 = -6.0547; gamma1 = 3.2758; gamma2 = 0.1711 | 1128.6 | 1138.5 |  | gamma0 = -6.6864; gamma1 = 2.2505; gamma2 = 0.1334 |
| fp2 | 1210.6 | 1223.9 |  | gamma0 = -4.756; gamma1 = 1.580; gamma2 = -0.858; gamma2 = 1.092 | 1130.3 | 1143.6 |  | gamma0 = -6.367; gamma1 = 2.033; gamma2= -0.194; gamma2 = 0.409 |
| gam | 1220.2 | 1235.0 |  | Intercept = -0.8159; s(time).1 = 1.2769; s(time).2 = 1.3707; s(time).3 = 3.0434; s(time).4 = 2.0028; k = 5 | 1141.08 | 1154.6 |  | Intercept = -1.1487; s(time).1 = 0.83; s(time).2 = 1.1944; s(time).3 = 2.7121; s(time).4 = 1.8616; k = 5 |
| pem |  |  | **1232.1** | Change point1 = 3.942; Change point2 = 8.245; Change point3 = 15.04;  lambda1 = 0.02278; lambda2 = 0.06946; lambda3 = 0.1224; lambda4 = 0.0521 |  |  | **1137.7** | Change point1 = 10.036;  lambda1 = 0.0152; lambda2 = 0.04075 |

PFS, progression-free survival; OS, overall survival; AIC, Akaike Information Criterion; BIC, Bayesian Information Criterion; WAIC, Watanabe-Akaike information critera; exp, exponent; lnorm, log-normal; llog, log-logistic; gam, gamma; ggam, gen-gamma; gomp, gompertz; rp, ryston-parmar spline model; rcs, restricted cubic splines; fp, fractional polynomials; pem, piecewise exponential model.

※：According to the AIC、BIC and Watanabe-Akaike information criteria, when the AIC、BIC and WAIC values are the smallest, the corresponding parameter distribution is the best fit. In this study, log-logistic distribution provided the best OS fit (both BC and ABC arms), whereas RP provided the best PFS fit (odds-2 for BC arm and harzed-3 for ABC arm) (Marked red in Table S3).

# Figure S1. The rebuilt extrapolated survival curves for BC arm and ABC arm.

1. Extrapolation of the fitted PFS curve for the ABC arm


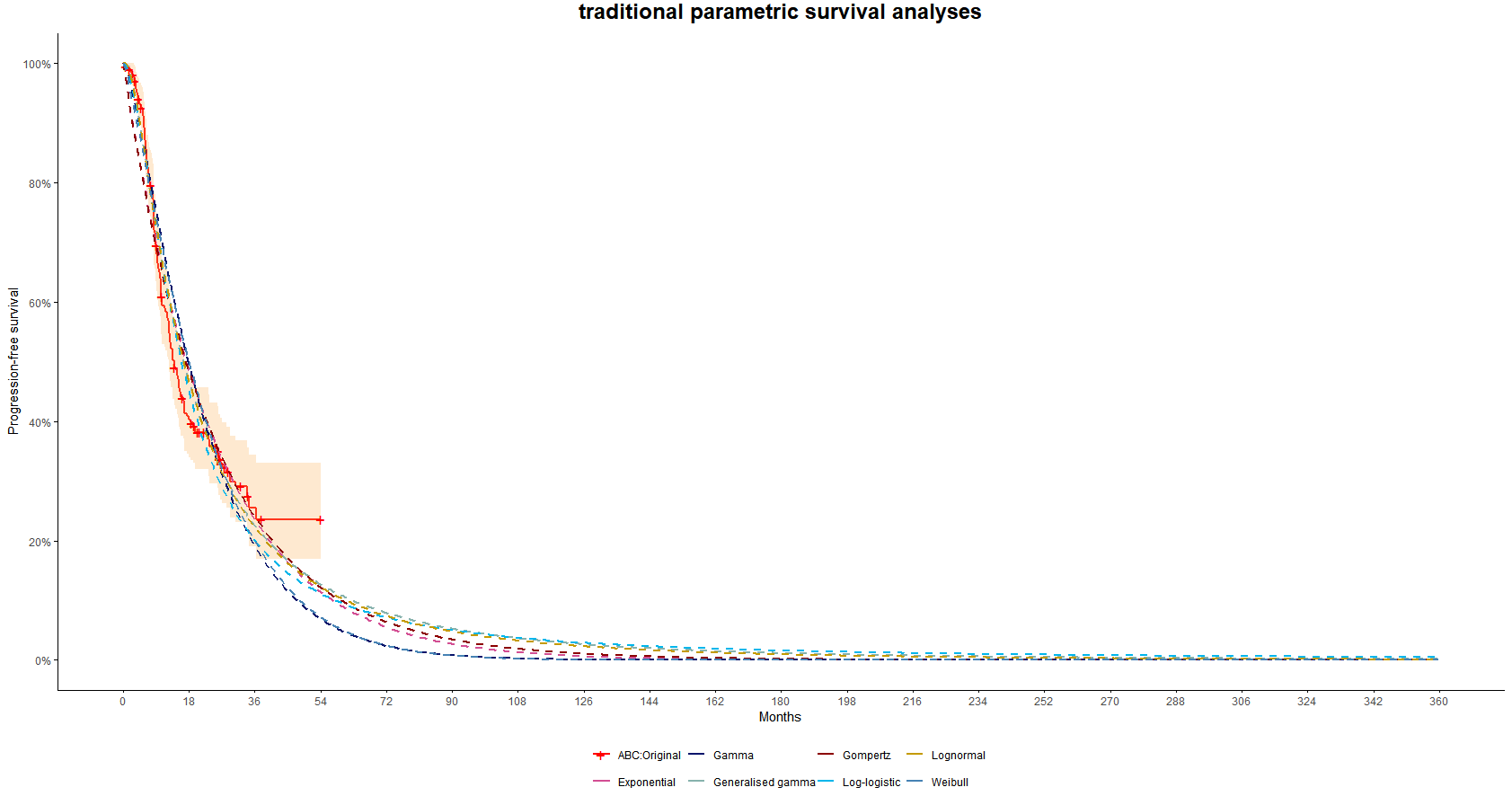

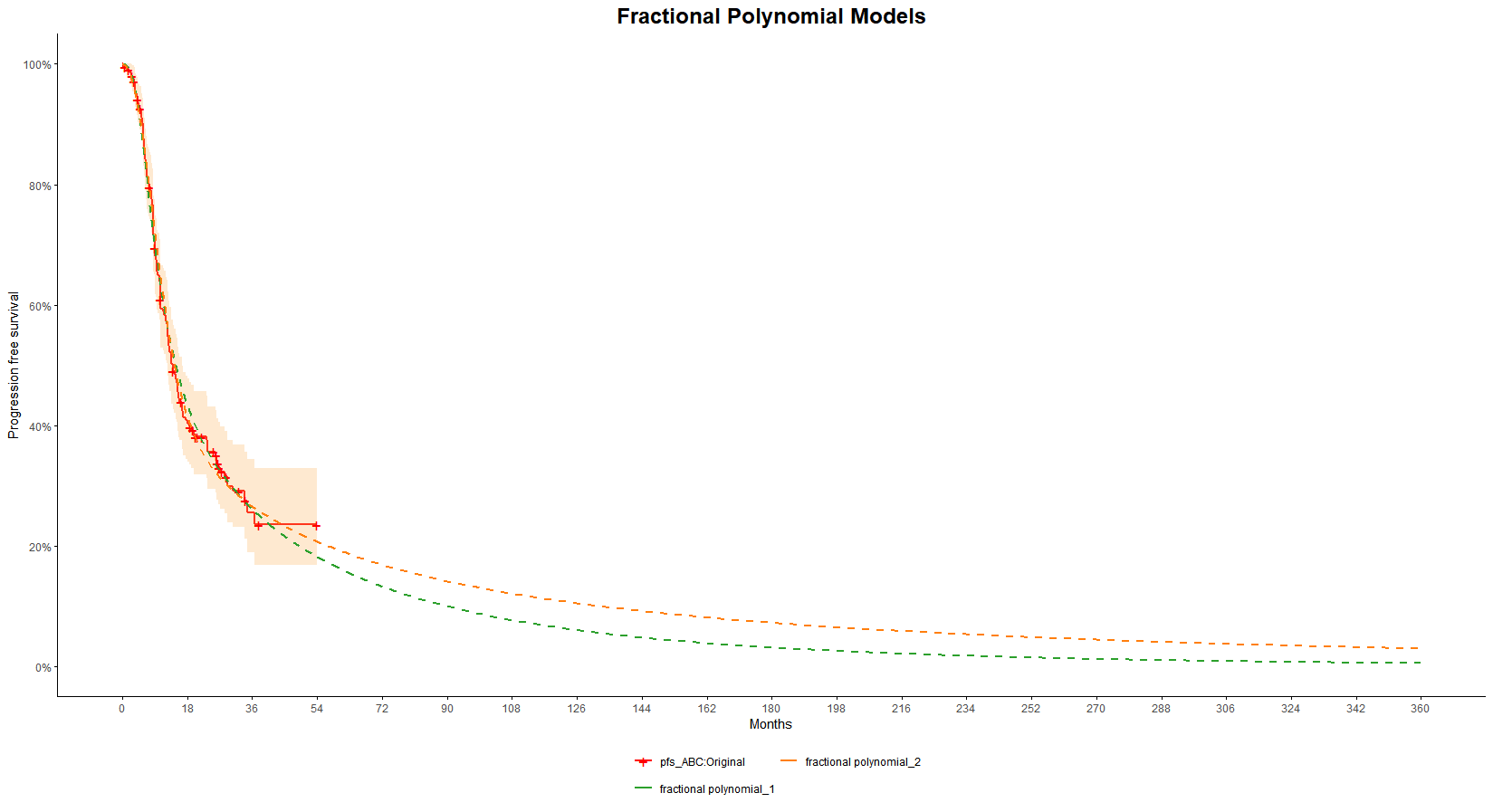

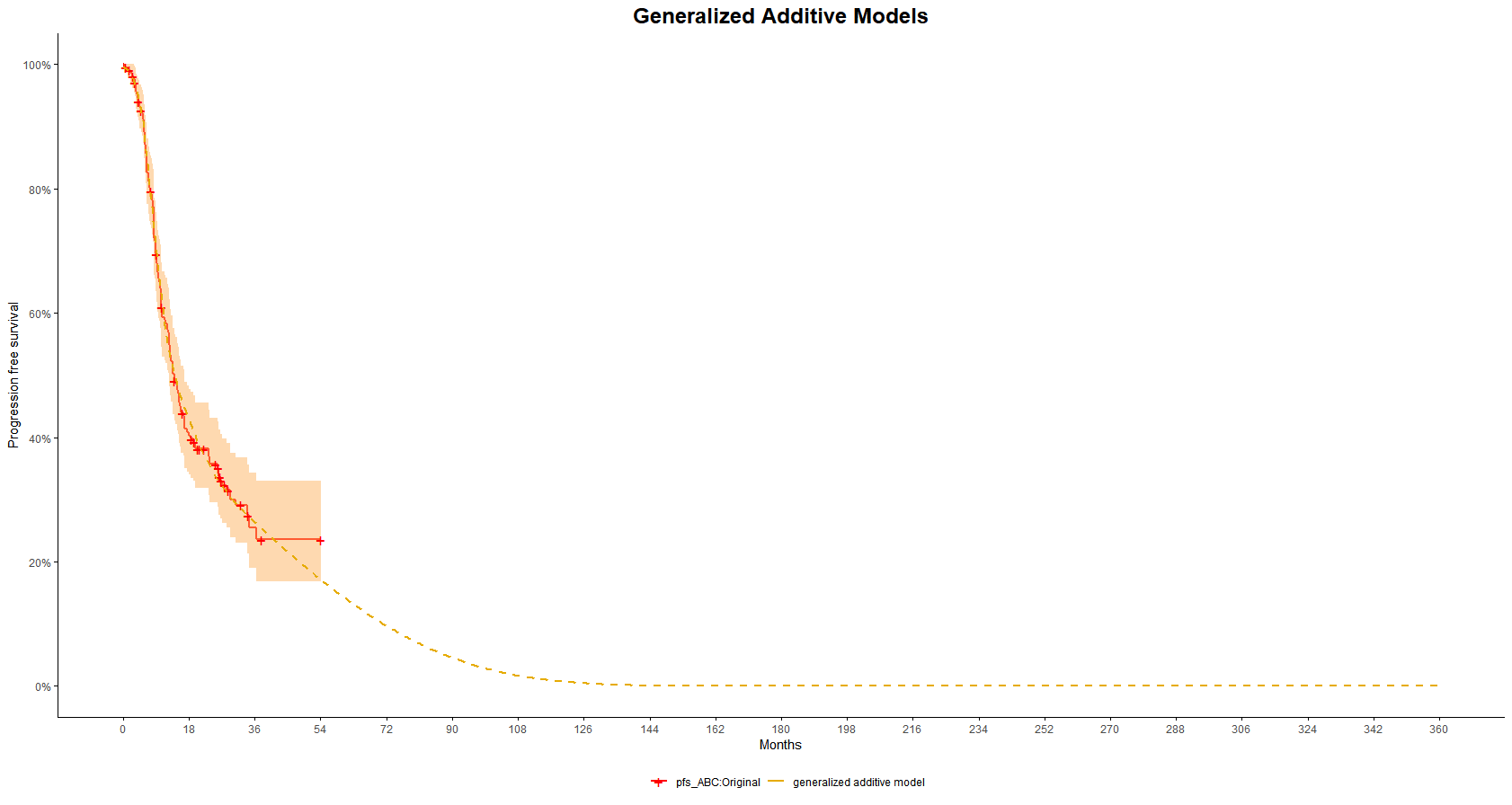

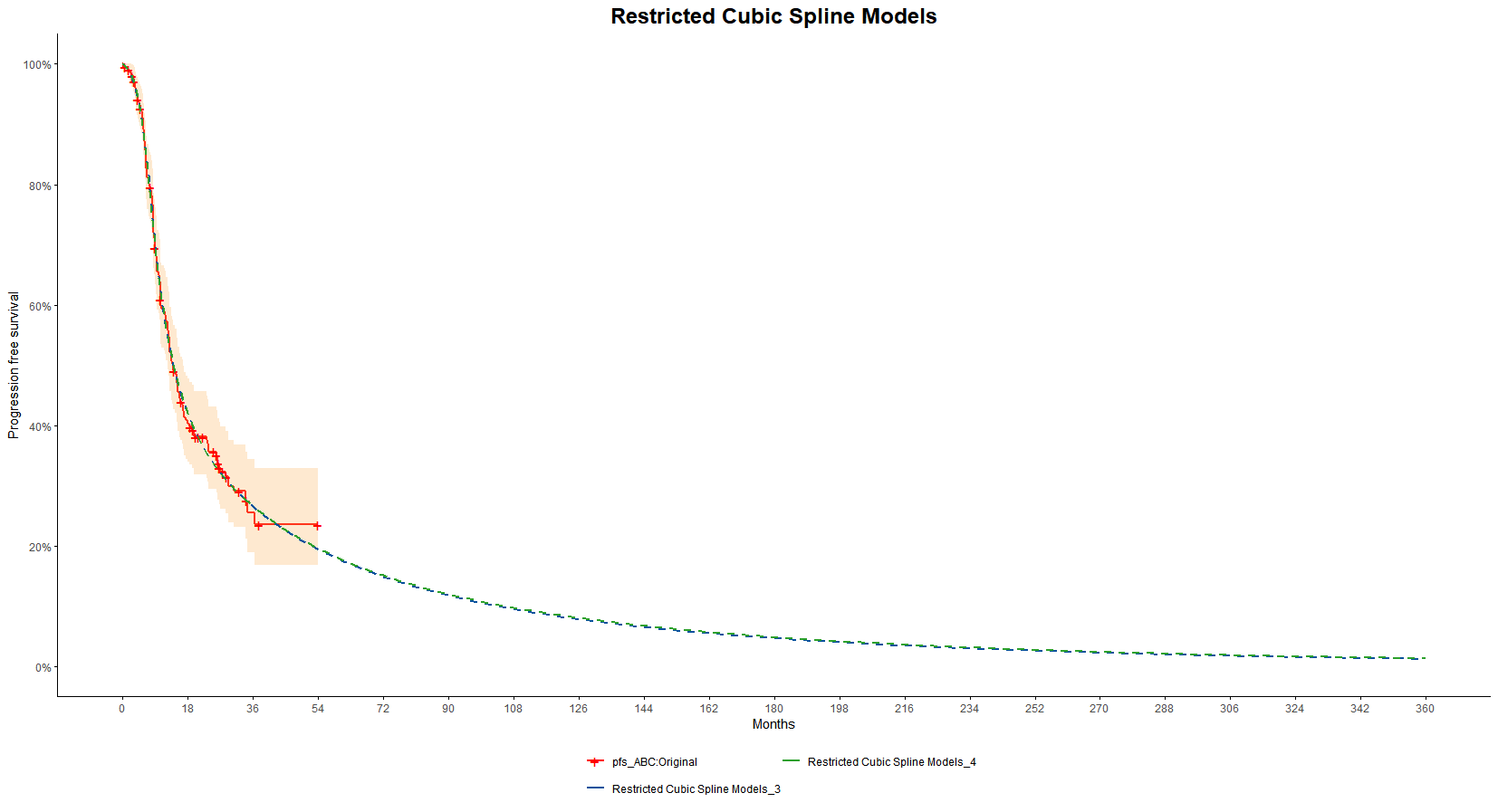

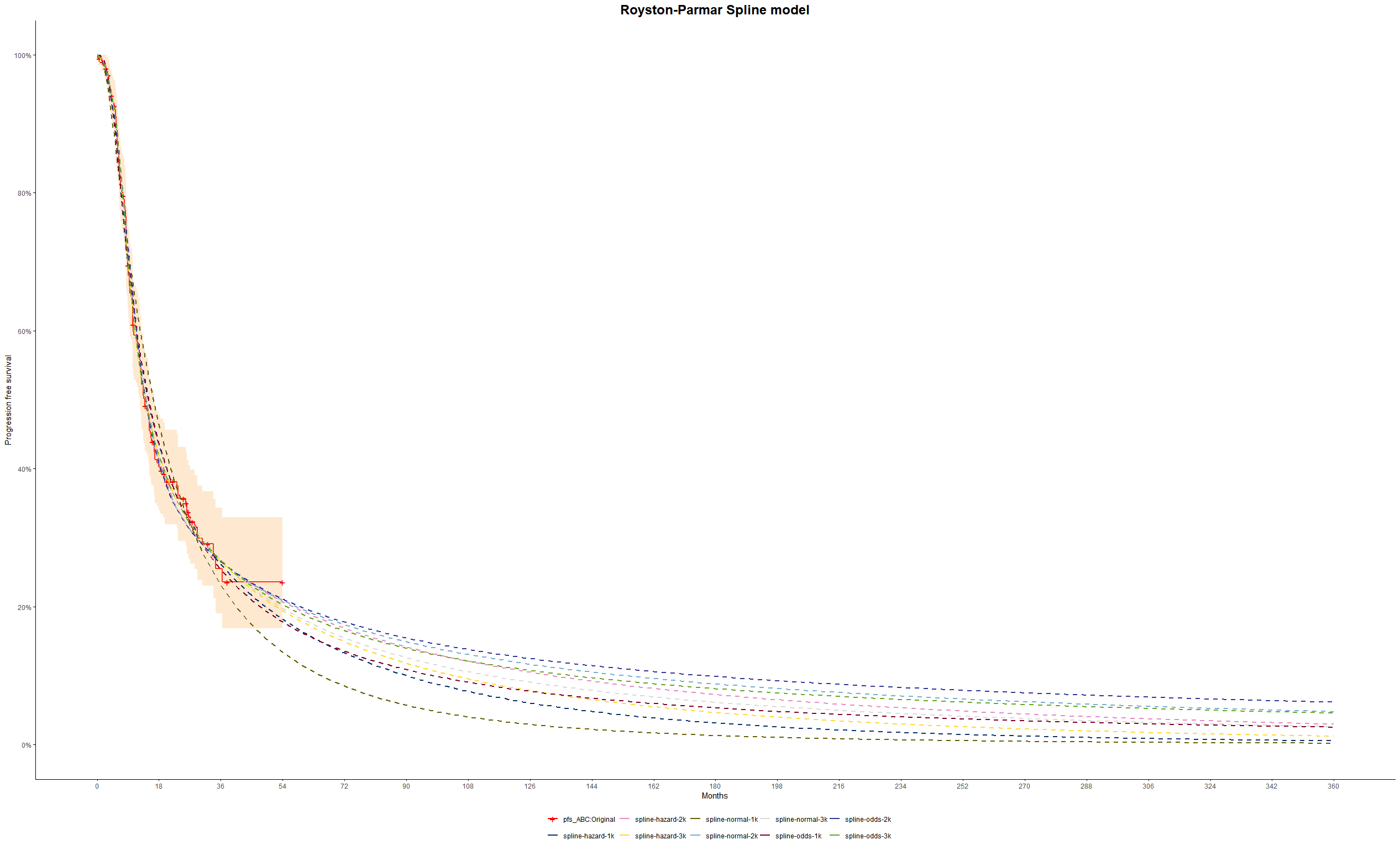

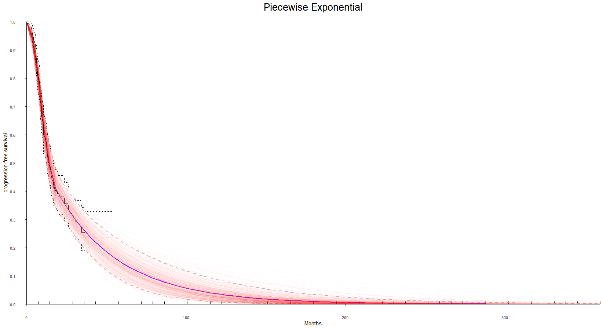


1. Extrapolation of the fitted PFS curve for the BC arm


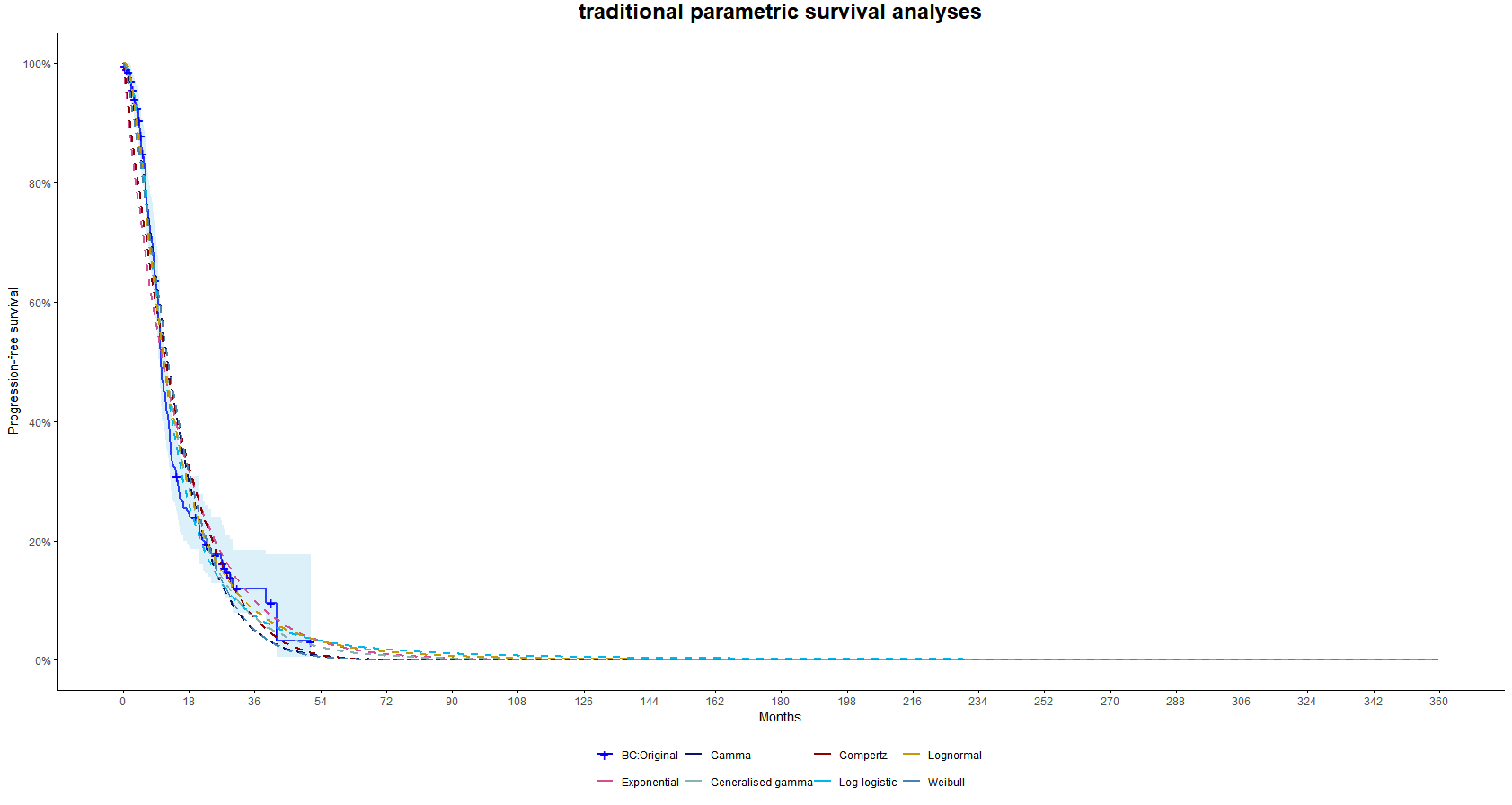

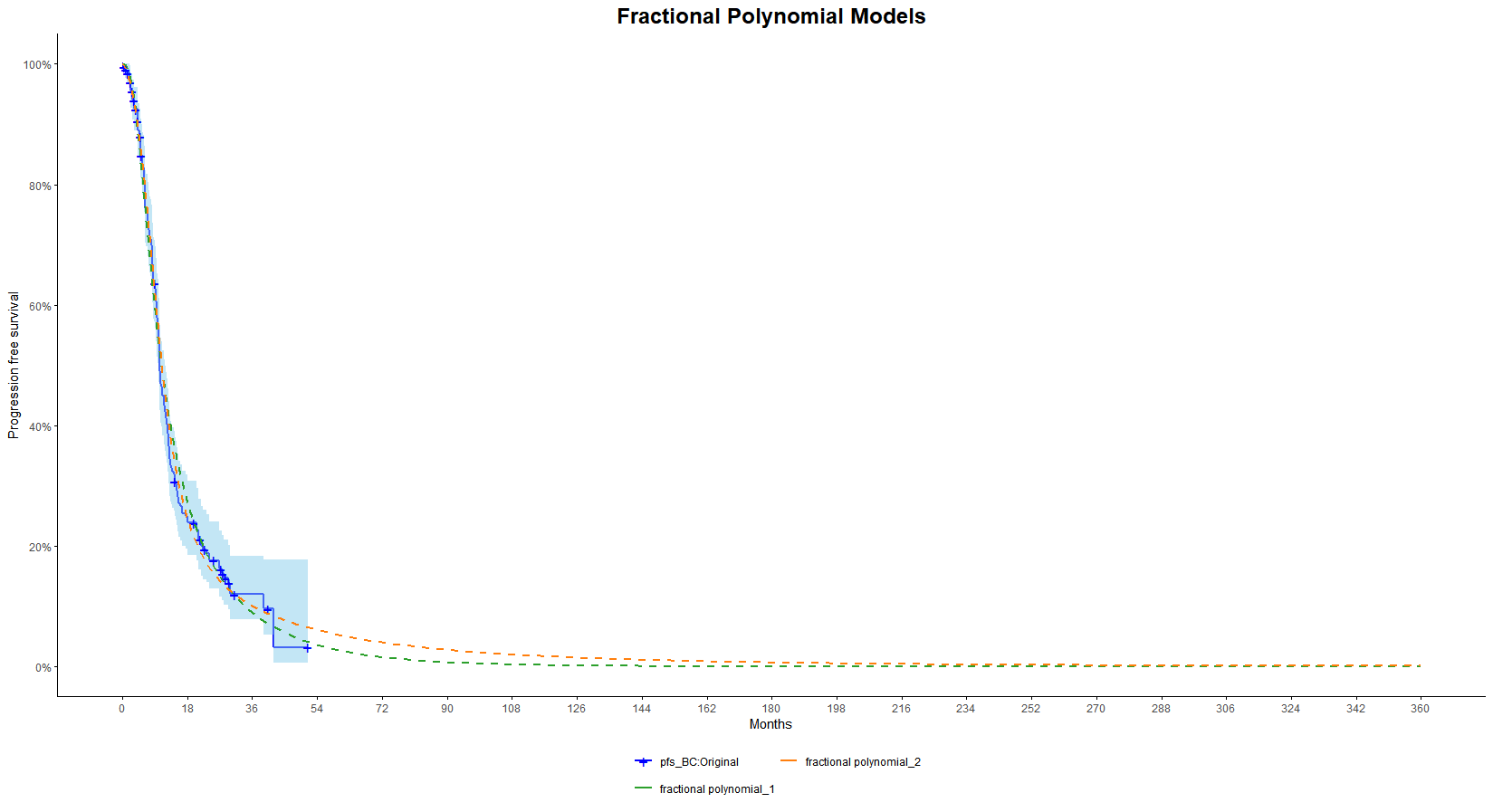

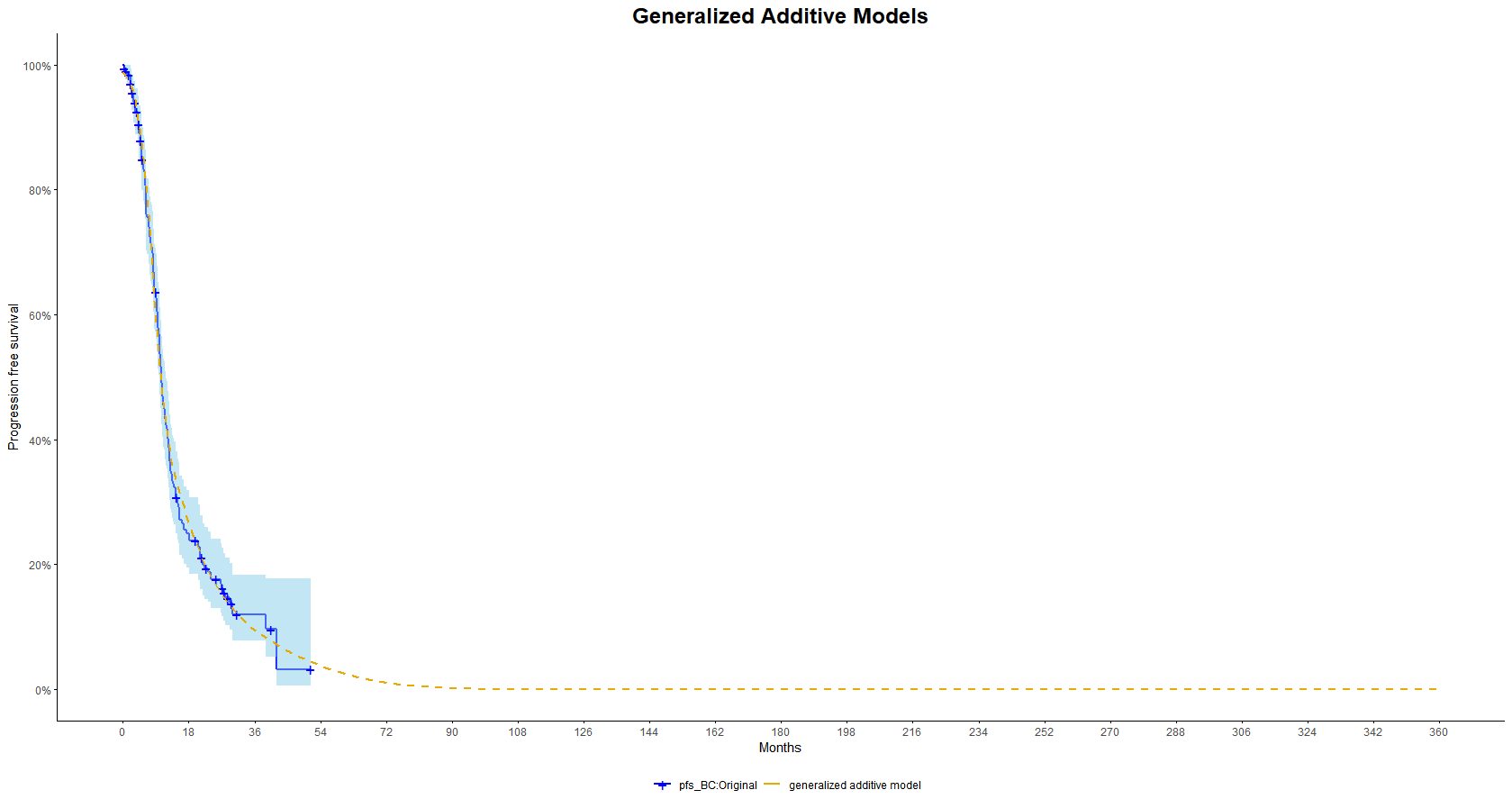

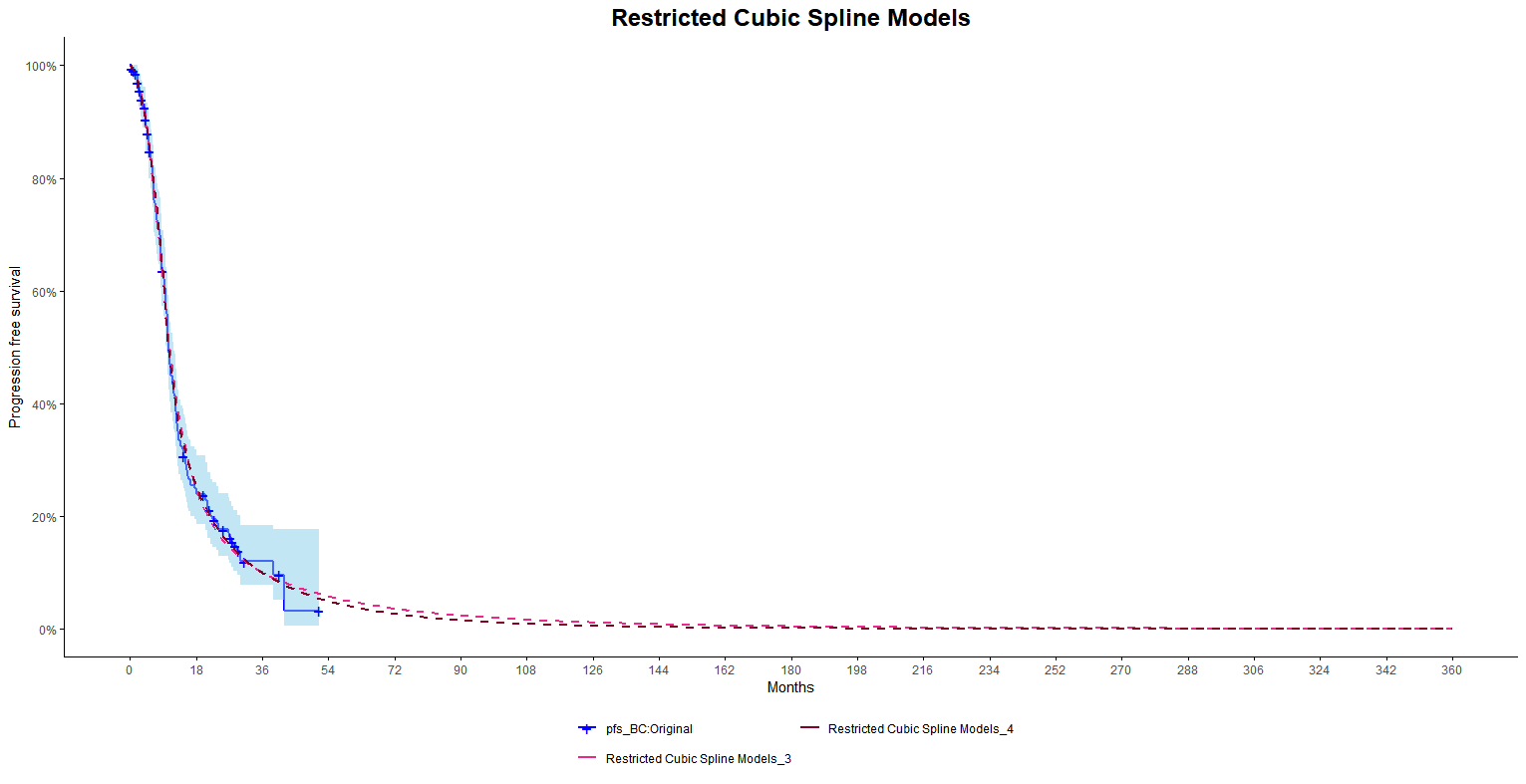

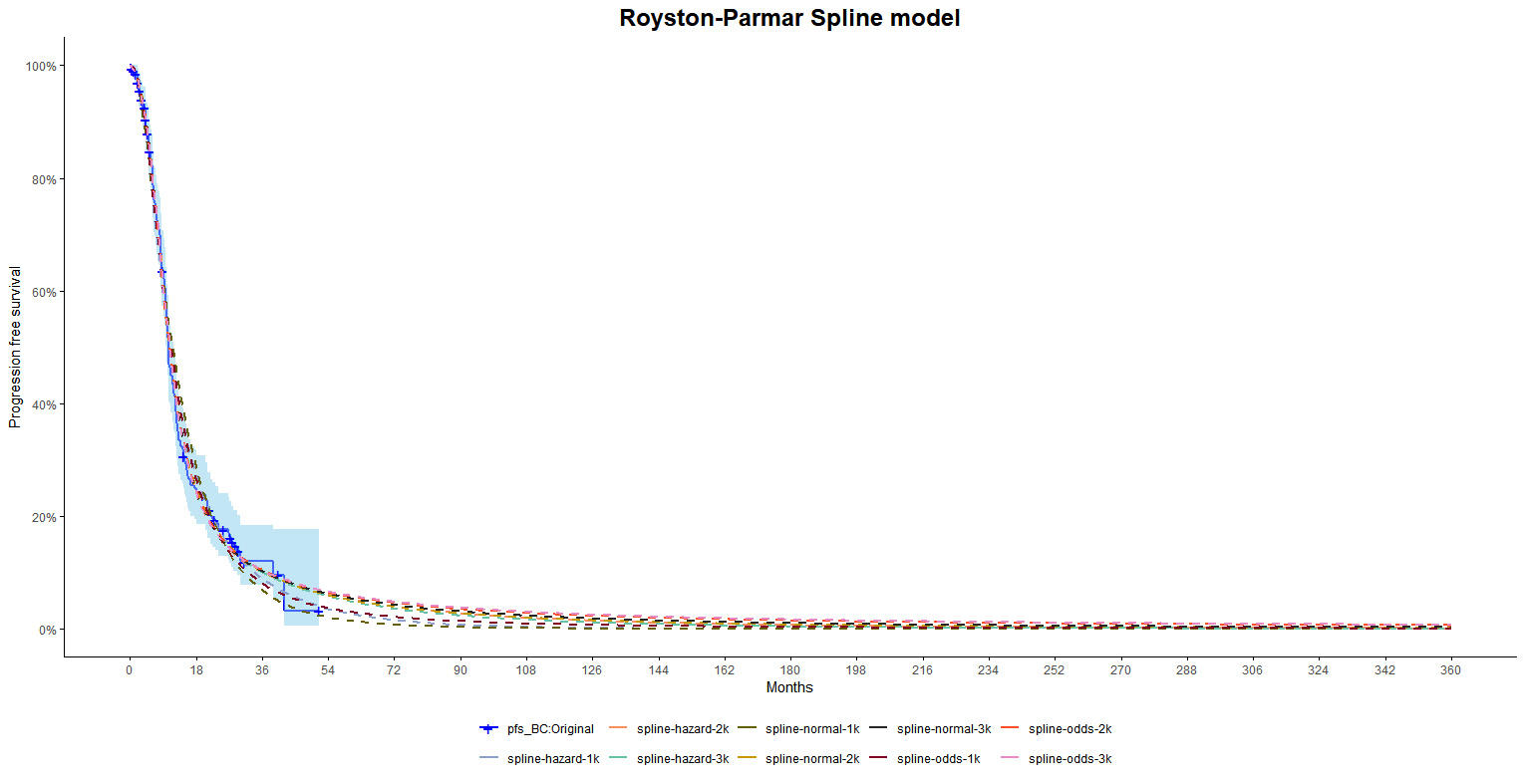

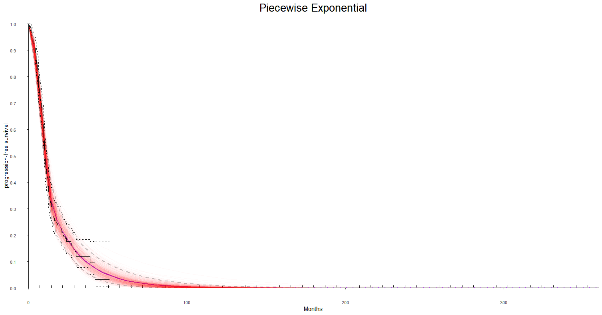


1. Extrapolation of the fitted OS curve for the ABC arm


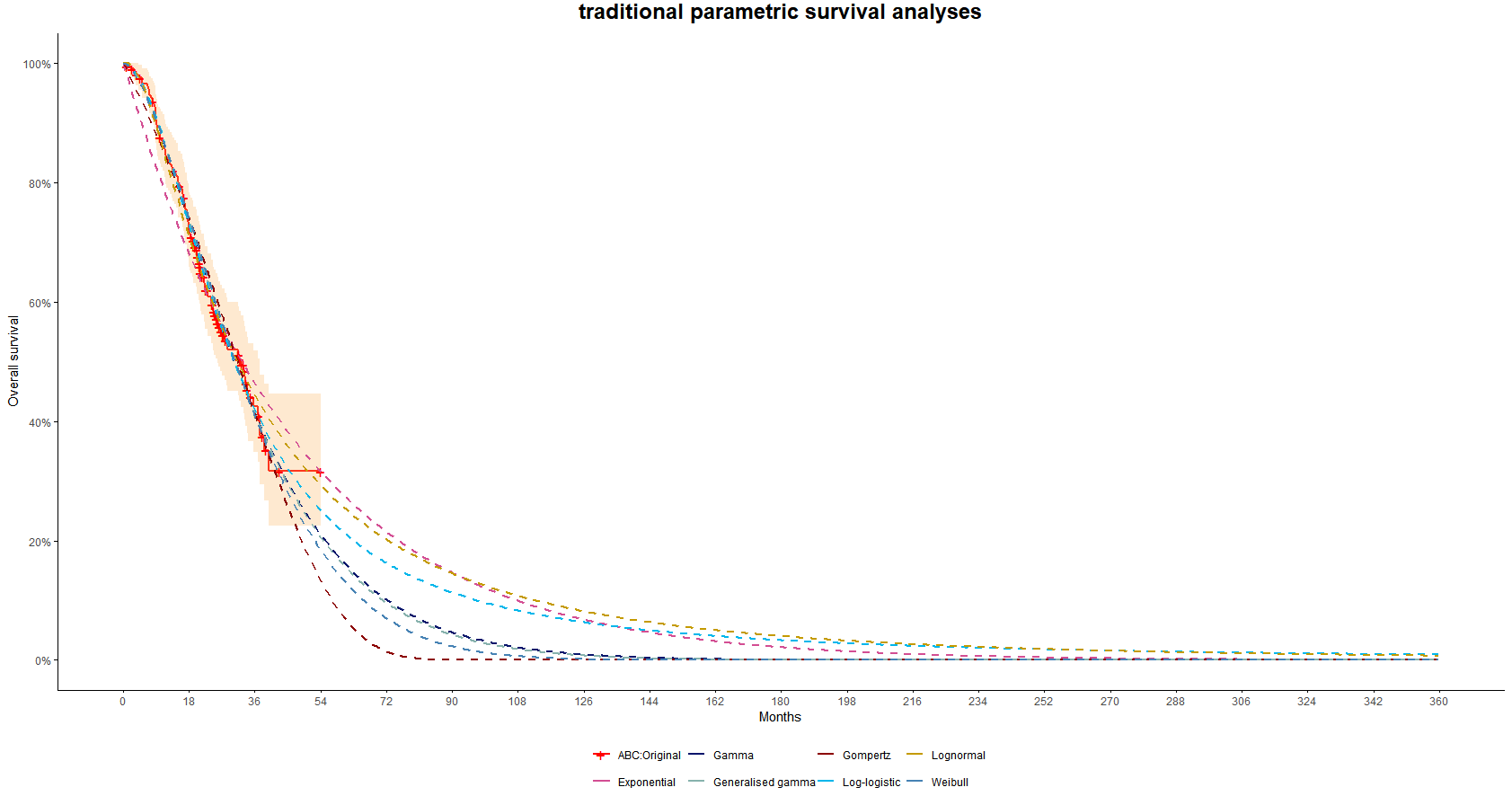

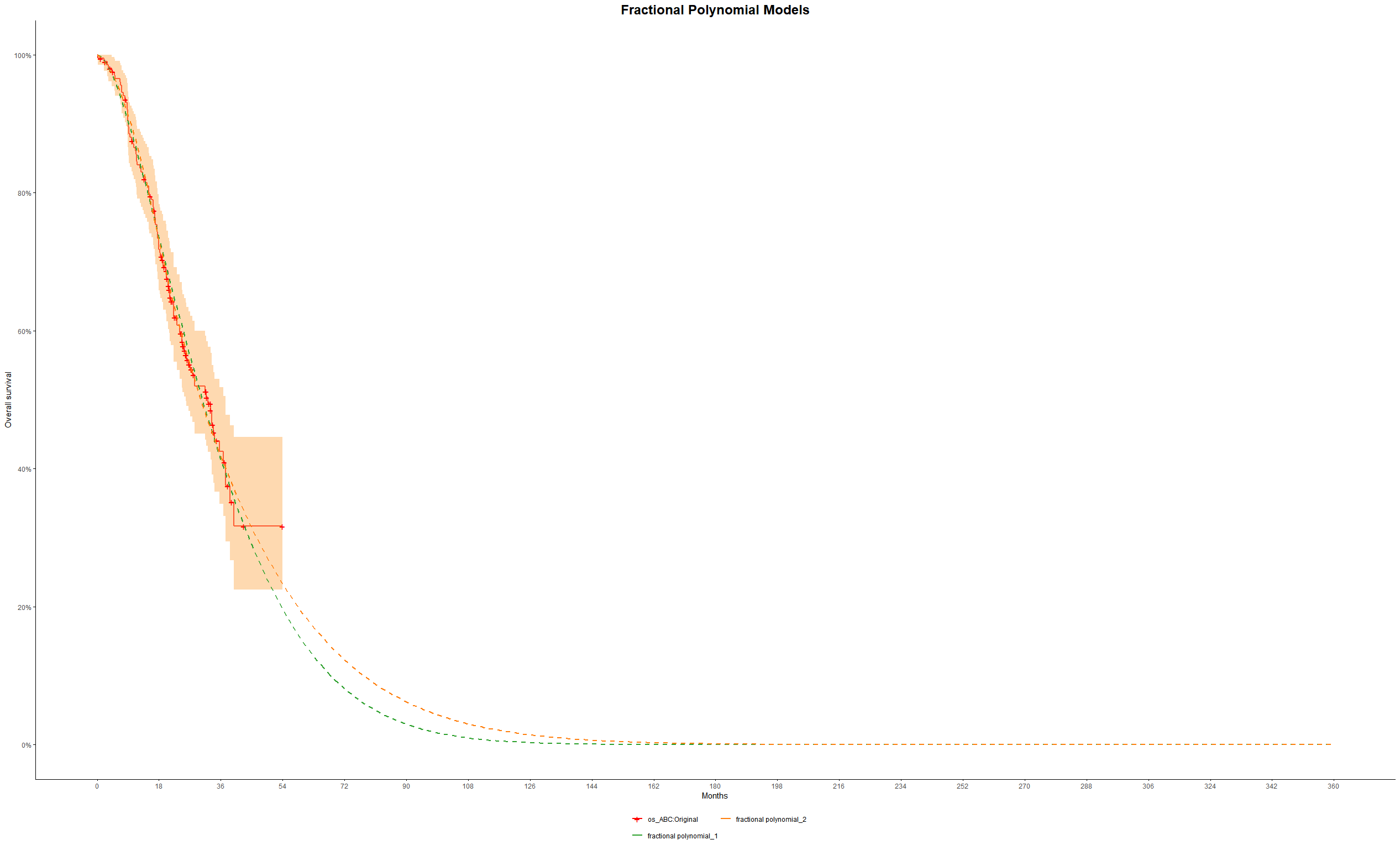

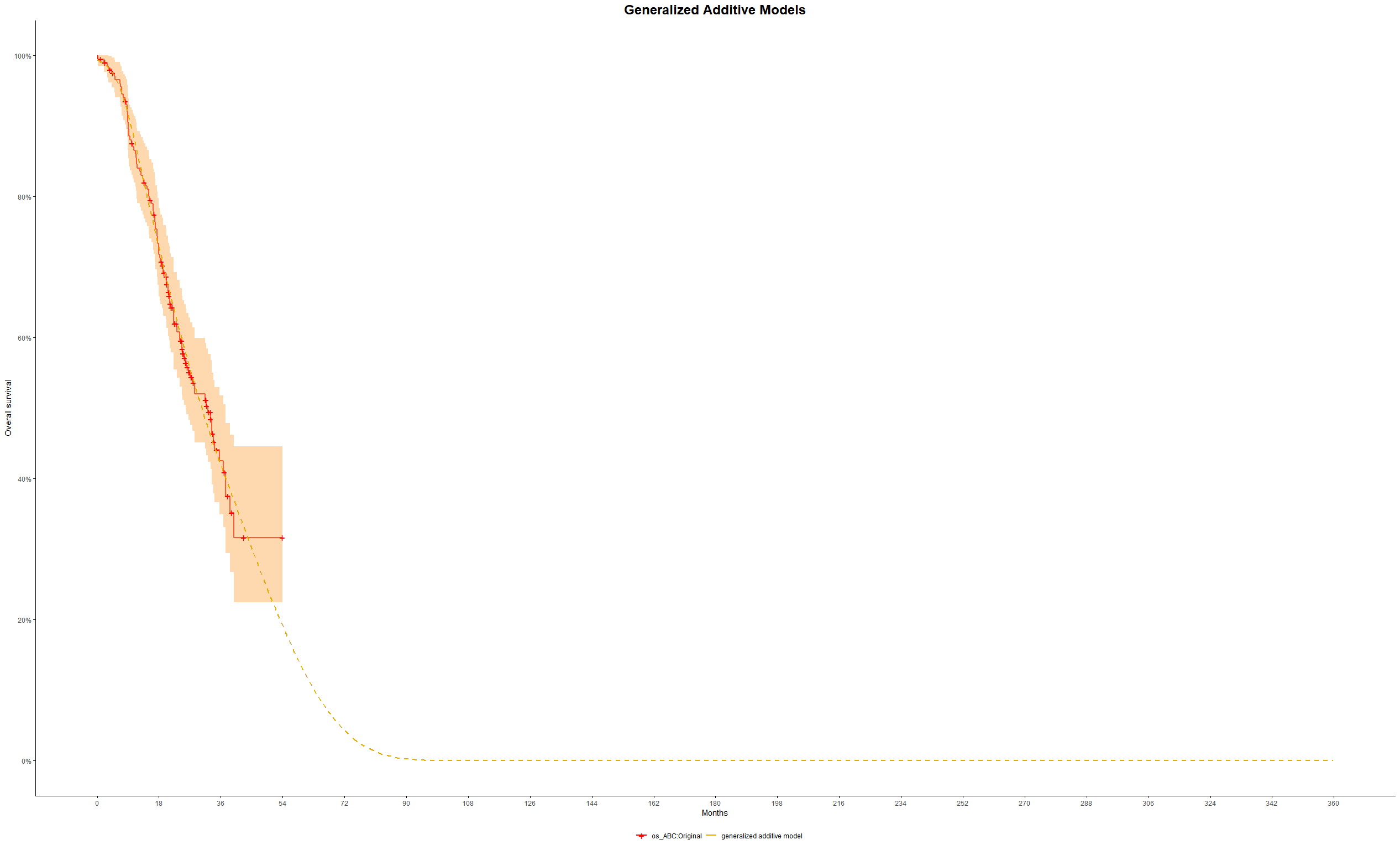

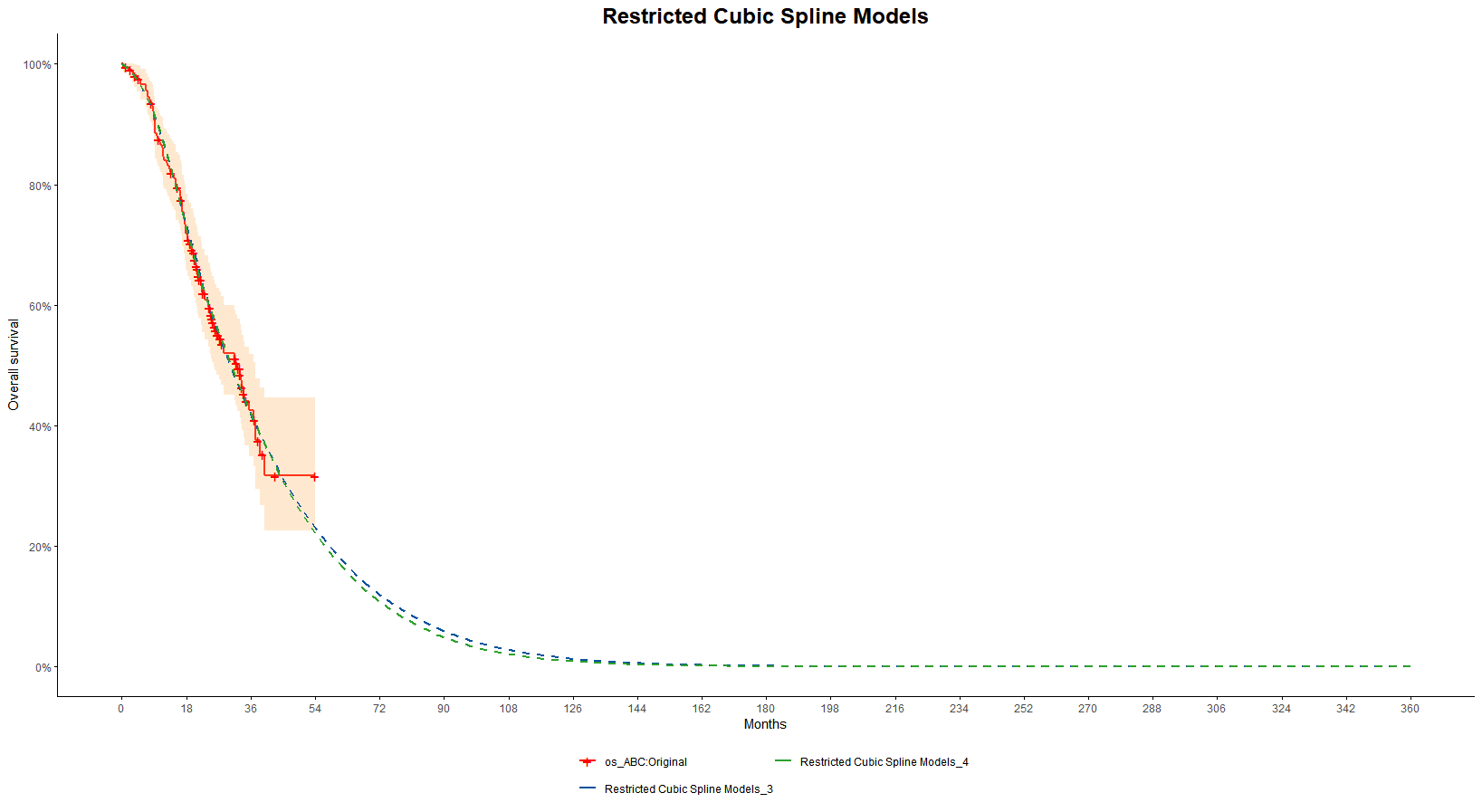

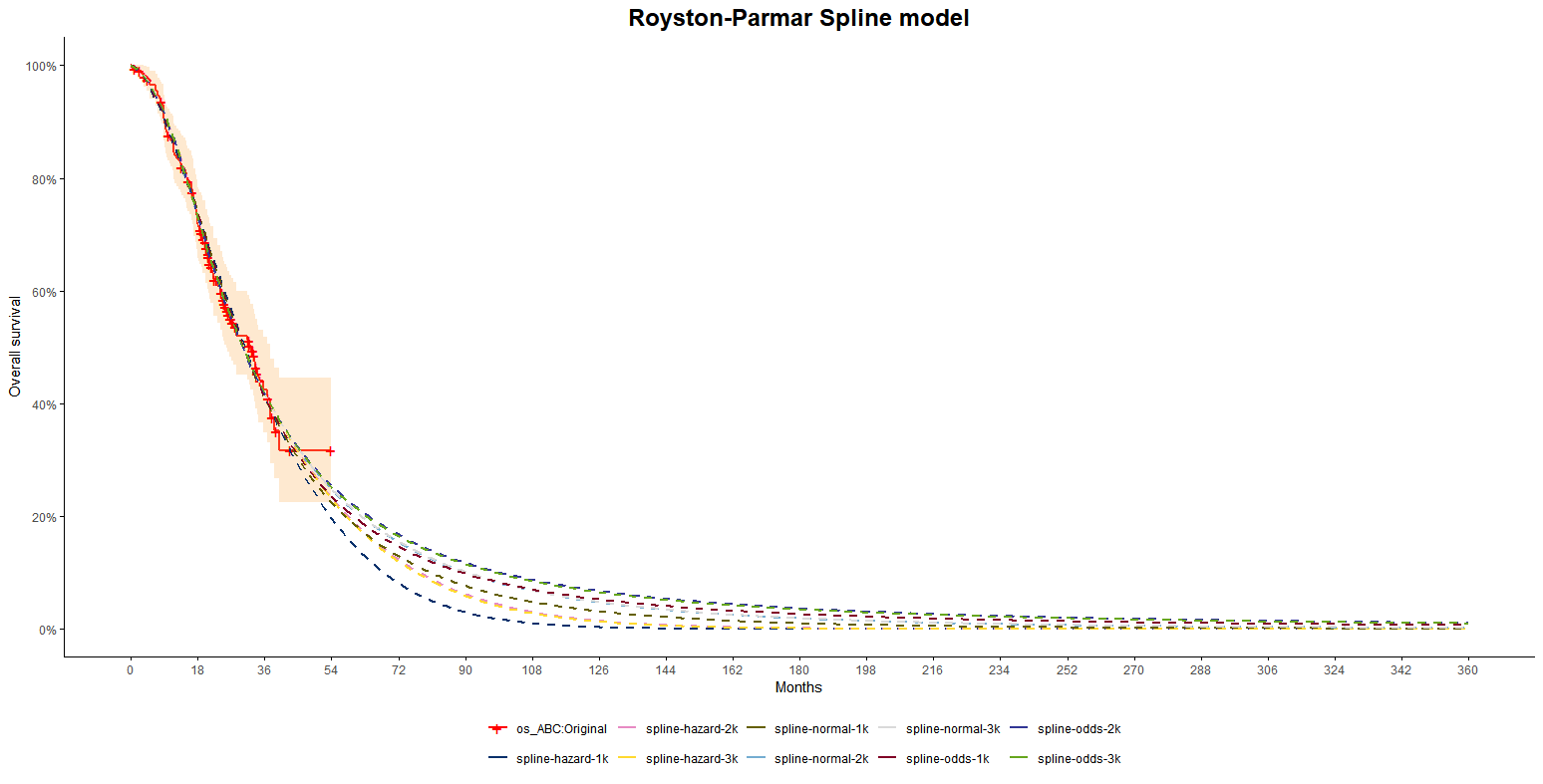

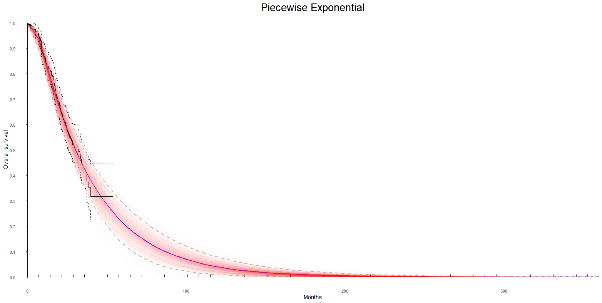


1. Extrapolation of the fitted OS curve for the BC arm


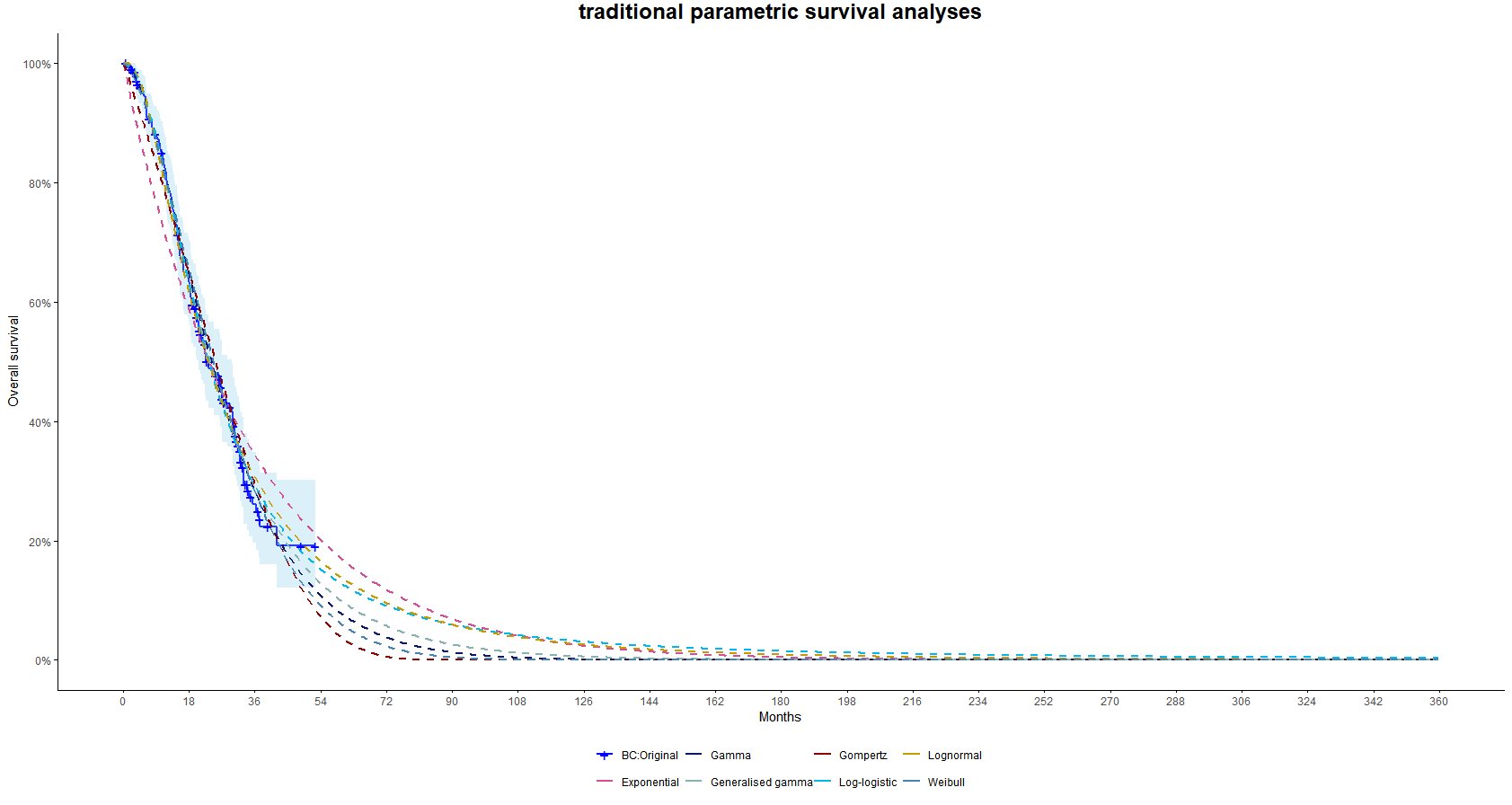

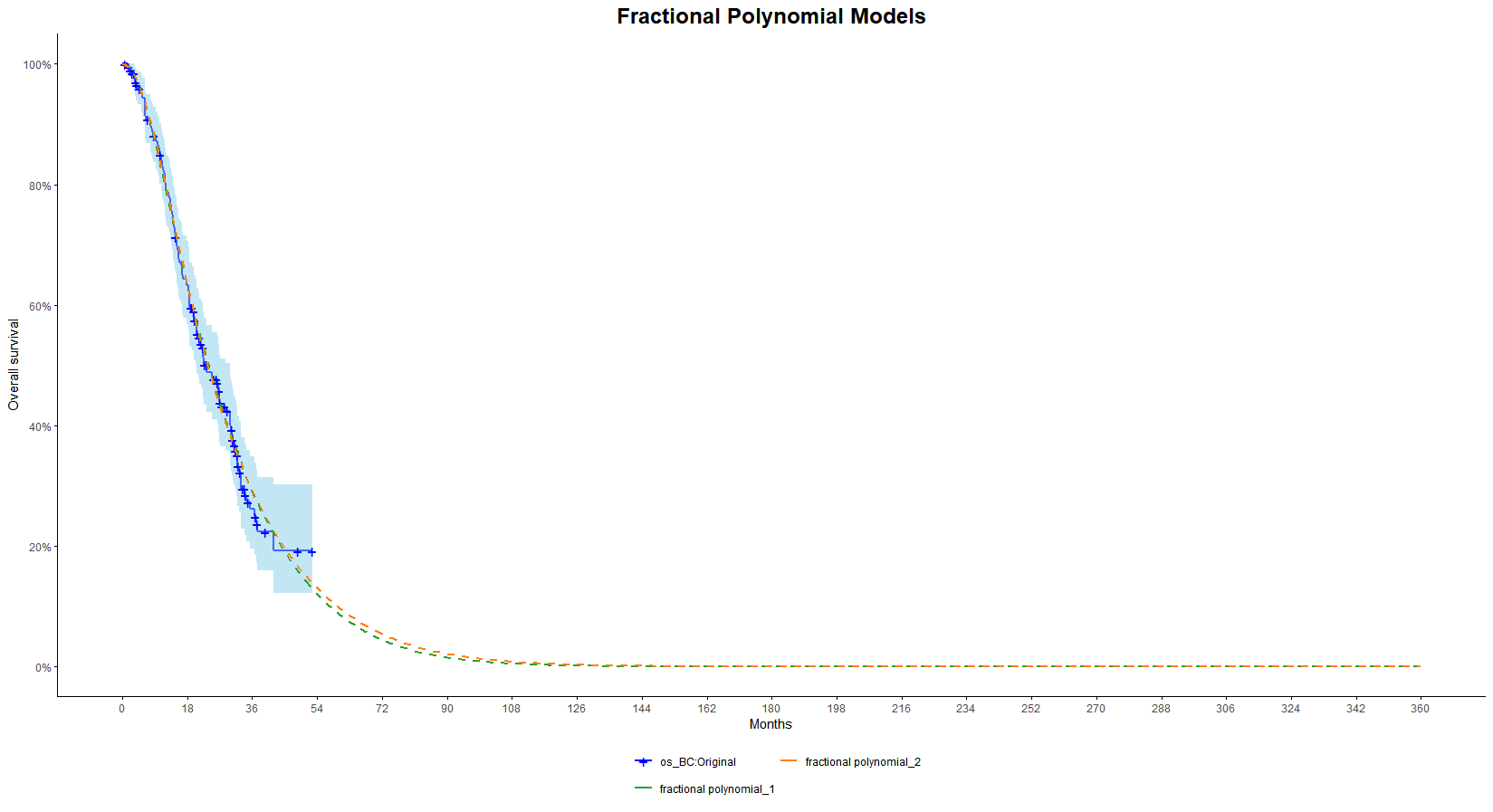

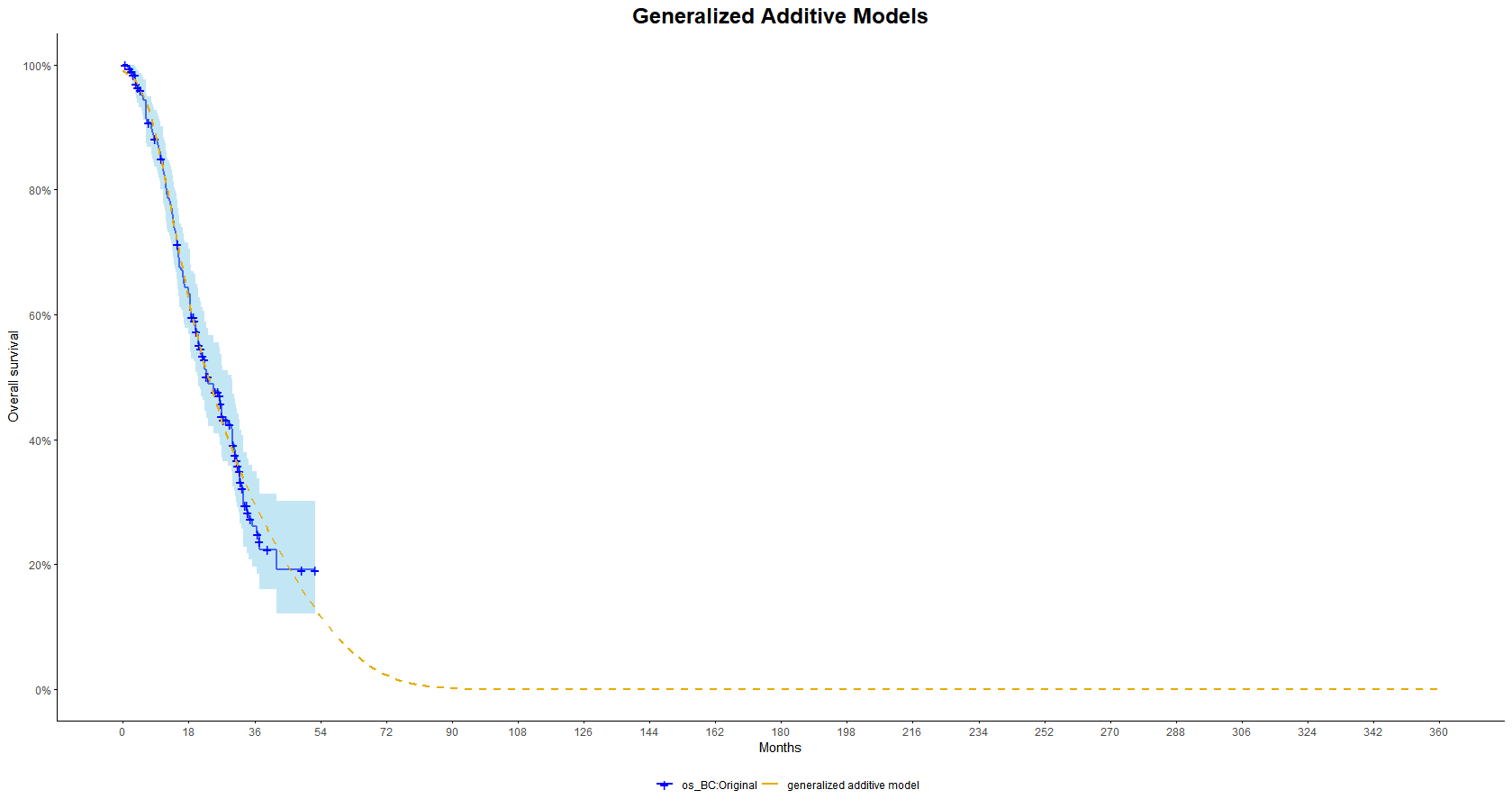

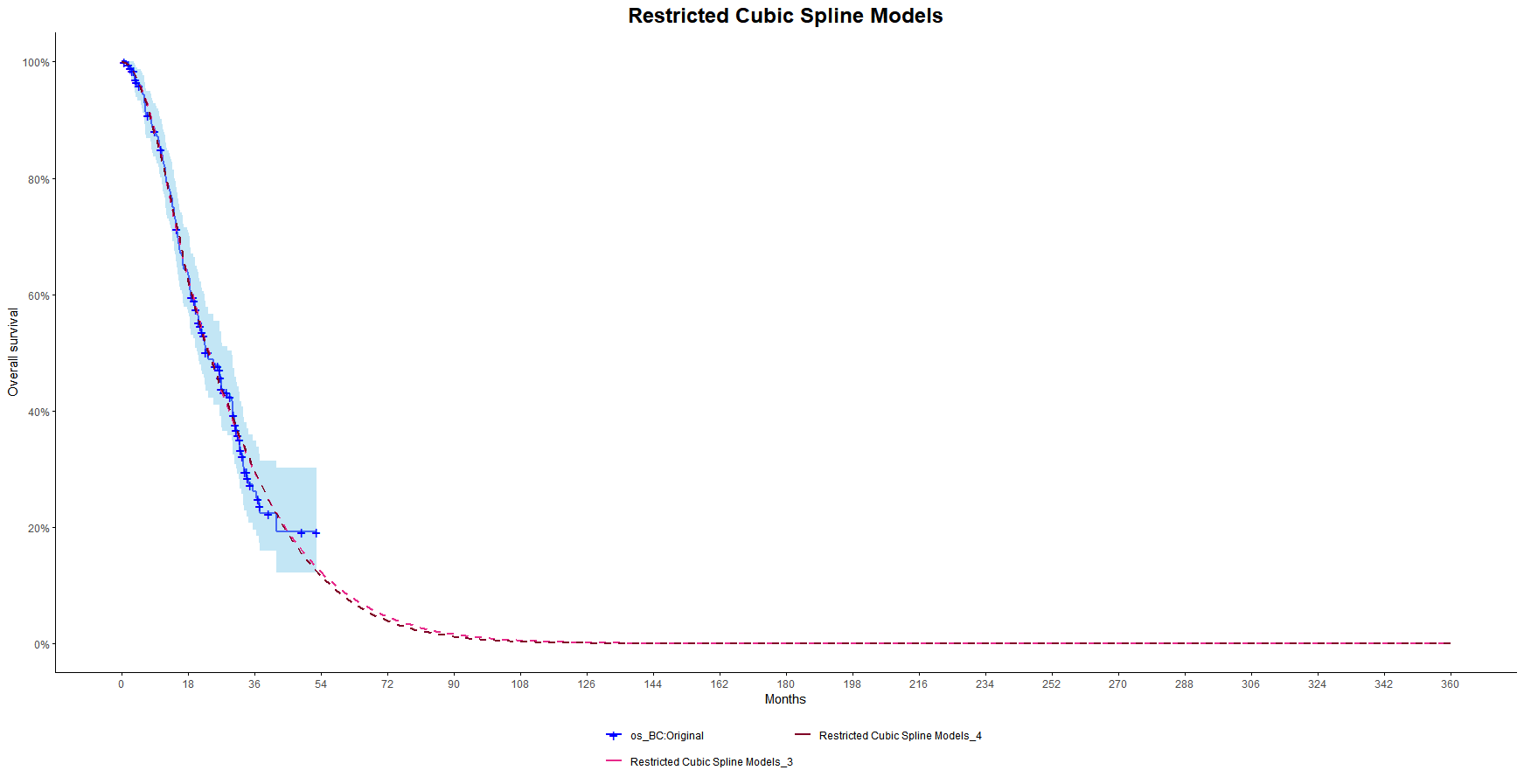

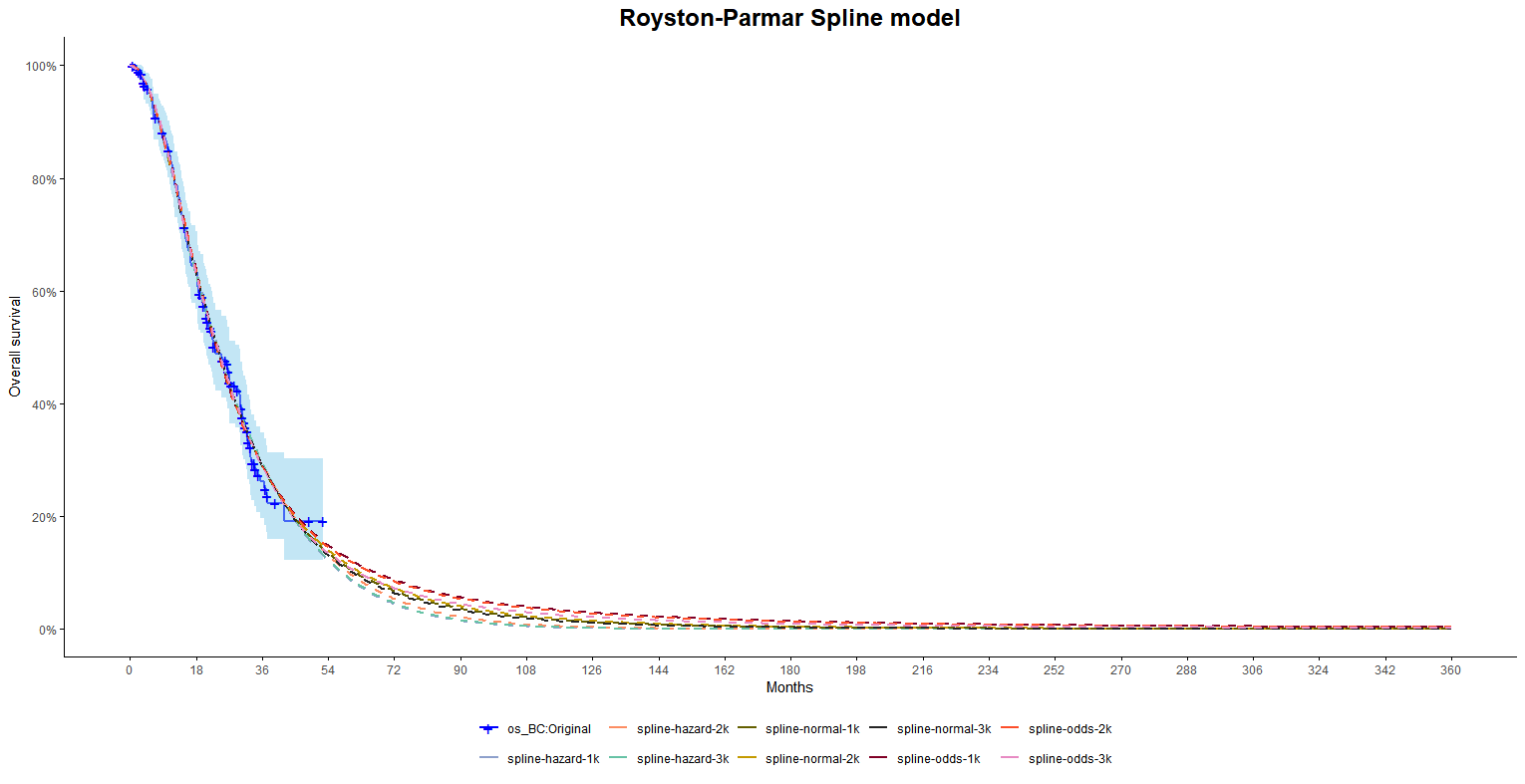

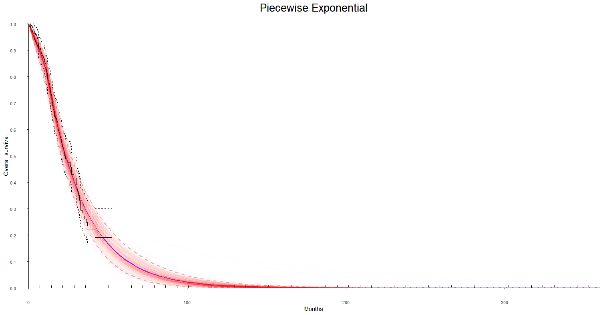

Supplement: Supplementary file 1 [file SupplementaryFile1.docx]
